# Deep Learning-Guided Reverse Translation Enhances Soluble Expression of Recombinant Proteins in *Escherichia coli*

**DOI:** 10.3390/ijms27115131

**Published:** 2026-06-05

**Authors:** Dong Yu, Nan Geng, Lin Fan, Yanmei Qin, Shangshang Sun, Hao Chen, Ruoyu Wang, Xiaoping Liao, Chun You

**Affiliations:** 1 College of Biotechnology, Tianjin University of Science and Technology, Tianjin 300457, China; yuddecho@gmail.com (D.Y.);; 2 Sino-Danish College, University of Chinese Academy of Sciences, Beijing 100049, China; 3 Tianjin Institute of Industrial Biotechnology, Chinese Academy of Sciences, Tianjin 300308, China; 4 University of Chinese Academy of Sciences, Beijing 100049, China

**Keywords:** deep learning, *Escherichia coli*, recombinant protein, codon optimization, soluble expression

## Abstract

Enhancing the soluble expression of heterologous proteins in chassis microorganisms is critical for fundamental biological research and synthetic biology-driven industrial applications. Current methods for designing DNA sequences to ensure high soluble expression often rely excessively on high-frequency codons while overlooking optimal codon context, leading to suboptimal outcomes. To address these limitations, we developed an integrated deep learning framework combining a synonymous codon generation (SCG) model and a gene expression level prediction (GELP) model. The SCG model captures codon usage patterns in *Escherichia coli* using large-scale genomic data, whereas the GELP model leverages gene expression data to prioritize sequences with high soluble expression potential. We validated our approach by optimizing the DNA sequences of two industrial enzymes, α-glucan phosphorylase (αGP) and isoamylase (IA), achieving significant and reproducible improvements in soluble expression (mean 12.2–16.9-fold, *n* = 3 and 2.6–3.4-fold, *n* = 4), confirmed by one-way ANOVA and one-sample *t*-tests. This study provides a useful tool for designing DNA sequences that confer high soluble expression and for understanding the relationship between DNA sequence and protein expression. Notably, SCG-GELP reveals a core-avoiding codon optimization strategy that substantially enhances soluble protein yield.

## 1. Introduction

Enzyme cost critically determines the economic viability of enzymatic processes at industrial scales. Established cost-reduction strategies include protein engineering to improve enzyme thermostability and activity [1], enzyme immobilization [2], and optimizing soluble protein expression [3]. Among these approaches, optimizing soluble protein expression permits straightforward monitoring via sodium dodecyl sulfate–polyacrylamide gel electrophoresis (SDS-PAGE), providing a rapid and cost-effective screening method. Consequently, enhancing the soluble expression of heterologous recombinant proteins in *E. coli* has emerged as a key focus area with substantial scientific and industrial relevance.

The soluble expression of heterologous recombinant proteins is influenced by multiple factors, including codon usage bias, host metabolic capacity, and culture conditions. Although expression yields can be improved by optimizing culture conditions [4,5]—such as adjusting medium composition, lowering incubation temperature, controlling expression rates with weak promoters, and co-expressing molecular chaperones [6]—the intrinsic DNA sequence remains a primary determinant of expression efficiency. It plays a pivotal role in regulating mRNA stability, translation kinetics, and soluble protein yield [7,8], and sequence optimization therefore exerts a greater impact on expression levels than external condition adjustments.

The process of generating a DNA sequence from a protein sequence is termed reverse translation. Because the genetic code is degenerate, a single protein sequence can be encoded by a vast number of potential DNA sequences [9]. To identify optimized DNA sequences that enhance heterologous soluble expression, codon optimization is employed, taking into account various factors such as codon usage bias, codon pair context, tRNA availability, GC content, ribosome binding sites (RBS), hidden termination codons, motif avoidance, restriction site removal, mRNA secondary structure, and hydrophilicity index. Numerous computational tools, including DNAWorks [10], JCat [11], OPTIMIZER [12], mRNA Optimizer [13], Gene Designer [14], Visual Gene Developer [14], COOL [15], and D-Tailor [16], have been developed to facilitate codon optimization by adjusting these factors. However, owing to the limited understanding of host cellular mechanisms, the expression levels of genes optimized using these traditional methods often fall short of expectations [17]. Recently, deep learning approaches have outperformed traditional methods in codon optimization by capturing complex nonlinear relationships and hidden patterns among expression-influencing factors, offering a promising alternative [18,19,20]. Accordingly, several deep learning-based codon optimization methods have been developed, including BiLSTM-CRF [21], ICOR [22], CodonBERT [23], CodonTransformer [24], and DeepCodon [25]. Nevertheless, the BiLSTM-CRF model was trained on a small dataset and fails to capture the relationship between DNA sequences and expression levels. ICOR lacks experimental validation and does not adequately model the sequence–expression relationship. CodonBERT, despite its advanced design, is limited by a small fine-tuning dataset comprising only three proteins and relies on normalized fluorescence values rather than actual expression levels, compromising its generalizability to industrial proteins.

To overcome these limitations, we present SCG-GELP, a novel deep learning framework that integrates de novo DNA sequence generation with soluble protein expression prediction, enabling robust expression optimization in *E. coli*. The SCG model employs a transformer-based encoder–decoder architecture trained on *E. coli* genome data to generate synonymous codon sequences that comply with host-specific usage patterns, whereas the GELP model combines support vector machine (SVM) [26], multi-layer perceptron (MLP), and logistic regression (LR) algorithms with multimodal input features to identify sequences predisposed to high soluble expression. This dual-model approach ensures that optimized sequences not only adhere to host-specific codon usage patterns but also encode expression-enhancing features overlooked by rule-based tools. We rigorously validated SCG-GELP through experimental testing of two industrially relevant enzymes. For α-glucan phosphorylase (αGP), the optimized sequences achieved 12.2–16.9-fold increases in soluble yield compared to the wild-type sequence, significantly outperforming the GenScript-optimized design. Similarly, the isoamylase (IA) variants achieved soluble protein fractions of 11.8–12.6%—approximately 2.8–3.0-fold higher than the GenScript-optimized sequence (4.2 ± 0.7%). These results demonstrate SCG-GELP’s ability to uncover non-obvious sequence determinants of expression while maintaining translational fidelity, offering an effective alternative to conventional codon optimization strategies.

## 2. Results

### 2.1. Overview of the SCG-GELP Framework

**Figure 1:**
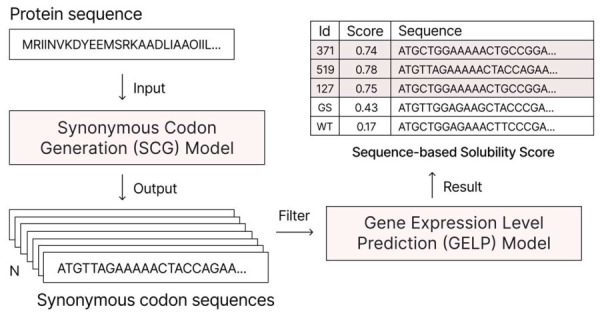
Overview of the SCG-GELP framework. Starting from a protein sequence, the SCG model generates candidate DNA sequences, which are then screened by the GELP model to select the sequence with the highest predicted soluble expression.

The SCG-GELP framework integrates two collaborative models to systematically optimize DNA sequences for high soluble expression in *E. coli* (Figure 1). The SCG model uses a Transformer-based encoder–decoder architecture to reverse-translate the input protein sequence into numerous high-quality candidate DNA variants. The encoder processes the protein sequence through amino acid embedding and multi-head self-attention layers to extract hierarchical features, while the decoder autoregressively generates codon sequences consistent with *E. coli* codon usage patterns. The generated DNA sequences are subsequently evaluated by the GELP model, which extracts sequence features through fine-tuned DNABERT-2 [27] and feeds them into an ensemble classifier (SVM, MLP, and LR). The sequence with the highest mean predicted probability of conferring high soluble expression is selected as the final output.

### 2.2. SCG Model Performance Evaluation

We trained the SCG model on the RefSeqE (Reference Sequence *E. coli*) dataset (98,855 protein-DNA sequence pairs). The training loss decreased steadily throughout optimization, whereas the test loss dropped rapidly during the first 10 epochs and subsequently plateaued at approximately 0.97–0.98. Using early stopping, the final test loss converged to 0.969.

**Figure 2:**
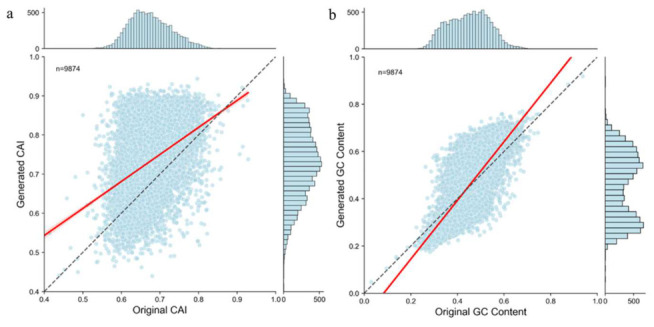
Performance metrics of the SCG model. (**a**) Codon Adaptation Index (CAI) distribution plot. (**b**) Distribution plot of guanine–cytosine (GC) content. The horizontal axis is the real sequence values, the vertical axis is the predicted sequence values, the dashed line is the coordinate axis angular bisector y = x, the red line is the scatter fit curve on the graph, and the histograms of kernel density statistics for the real and predicted sequences are shown at the top and right of the graph, respectively.

To evaluate the performance of the SCG model, we employed a decoding strategy integrating beam search and pruning operations to generate large batches of synonymous codon sequences for proteins in the test set. The top-scoring predicted sequences were selected, and the Codon Adaptation Index (CAI) [28] and guanine–cytosine (GC) content were calculated and compared against the corresponding real sequences in the test set, as shown in Figure 2. Figure 2a presents the CAI distribution between real and predicted sequences, with the horizontal axis representing real sequence values, the vertical axis representing predicted sequence values, the dashed line indicating the diagonal y = x, and the red line showing the fitted curve for the scatter plot; kernel density histograms for the real and predicted sequence CAIs are displayed at the top and right of the panel, respectively. Figure 2b shows the corresponding GC content distribution, using the same layout. For the CAI distribution, we observed that the CAI values of most sequences were improved after optimization, indicating that the model tends to favor codons with higher usage preference during sequence design. Moreover, the peak CAI value of the optimized sequences remained approximately 0.9, suggesting that while the model preferentially selects high-frequency codons, it does not extremize all codons to the most preferred option; instead, it generates rare codons that retain functional value [29]. Regarding GC content (Figure 2b), sequences with originally low GC content tended to be further reduced after optimization, whereas those with originally high GC content tended to increase, producing two distinct peaks in the post-optimization density histogram. The GC content of most optimized sequences fell within the range of 0.25 to 0.7. Collectively, the CAI and GC content distributions demonstrate that the SCG model effectively elevates CAI and optimizes GC content during synonymous codon generation.

### 2.3. GELP Model Performance Evaluation

**Figure 3:**
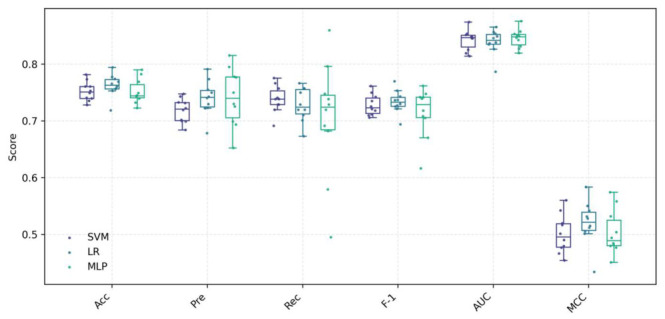
Ten-fold cross-validation performance of the Support Vector Machine (SVM), Multilayer Perceptron (MLP), and Logistic Regression (LR) classifiers on the Northeast Structural Genomics DNA (NESG-DNA) dataset.

The GELP model integrates DNABERT-2 for feature extraction and three machine learning classifiers (SVM, MLP, and LR) to predict whether a DNA sequence will yield high soluble expression. We fine-tuned DNABERT-2 on the NESG-DNA (Northeast Structural Genomics) dataset using default training parameters; after 1400 training steps, the model achieved a test loss of 0.5868 and an accuracy of 70.79%. In addition, we evaluated multiple performance metrics for each of the three classifiers using ten-fold cross-validation on the NESG-DNA (Northeast Structural Genomics) dataset. The average accuracy across all three algorithms exceeded 75%, with SVM achieving the highest accuracy at 81.17% (Figure 3).

### 2.4. Experimental Verification Results

To rigorously evaluate the SCG-GELP framework, we conducted systematic expression tests using two industrially relevant enzymes: α-glucan phosphorylase (αGP) from *Thermotoga maritima* and isoamylase (IA) from *Sulfolobus tokodaii* (see Supplementary Table S4 for detailed protein information). αGP catalyzes the reversible phosphorylation of α-glucan and serves as a key enzyme in numerous in vitro synthetic enzymatic biosystems that utilize α-glucan to produce hydrogen [30], electricity [31], and inositol [32]. IA hydrolyzes α-1,6-glucosidic branch linkages in glycogen and amylopectin, yielding amylodextrin for complete utilization of branched α-glucan [33]. The experimental design compared protein expression levels among wild-type sequences, GenScript-optimized sequences, and our SCG-GELP-optimized variants (Opt-1 and Opt-2) in an *E. coli* BL21 (DE3) expression system.

**Figure 4:**
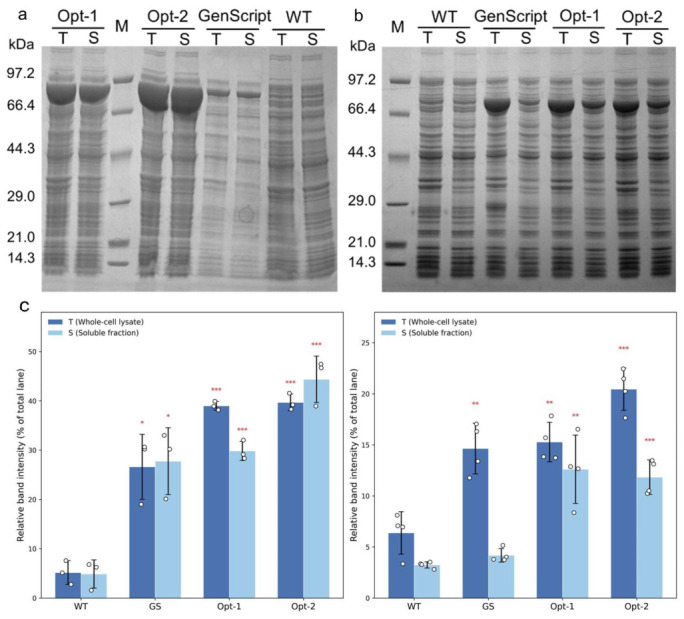
Experimental validation across independent biological replicates. (**a**,**b**) Representative SDS-PAGE gels for αGP ((**a**), representative of *n* = 3) and IA ((**b**), representative of *n* = 4). Lane M, protein molecular weight marker; Opt, DNA sequences optimized using SCG-GELP; GenScript (GS), GenScript-optimized DNA sequences; WT, wild-type sequences. T, whole-cell lysate; S, soluble fraction after centrifugation. (**c**) Quantitative analysis of relative band intensity (% of total lane signal). Bars represent mean ± SD; circles denote individual replicate values. Statistical significance was assessed by one-way ANOVA followed by Bonferroni-corrected post hoc *t*-tests comparing each optimized variant to the wild-type (WT) control. * *p* < 0.05, ** *p* < 0.01, *** *p* < 0.001. αGP data are from three independent experiments; IA data are from four.

To confirm the quantitative reproducibility of SCG-GELP-mediated expression enhancement, we performed three independent biological replicates for αGP and four for IA. For each replicate, cells harboring wild-type (WT), GenScript-optimized (GS), or SCG-GELP-optimized (Opt-1 and Opt-2) constructs were cultured, induced, and processed independently. Whole-cell lysates (T) and soluble fractions (S) were analyzed by SDS-PAGE, and protein band intensities were quantified using ImageJ Gel Analysis (Figure 4a,b).

Quantitative analysis revealed striking differences among the tested constructs (Figure 4c, Table 1). For αGP, one-way ANOVA of the relative band intensity (% of total lane signal) showed significant group effects in both whole-cell lysate (F = 58.98, *p* < 0.0001) and soluble fraction (F = 39.82, *p* < 0.0001). Bonferroni-corrected post hoc comparisons demonstrated that both Opt-1 and Opt-2 significantly outperformed WT in total expression (39.0 ± 0.9% and 39.7 ± 1.7% vs. 5.2 ± 2.4%, respectively; *p* < 0.001) and soluble yield (29.8 ± 1.9% and 44.4 ± 4.7% vs. 4.9 ± 2.9%; *p* < 0.001). GS also showed significant improvement over WT (*p* < 0.05).

Fold-change analysis (intra-replicate normalization to WT) confirmed that Opt-1 and Opt-2 achieved 14.5 ± 2.9-fold and 15.1 ± 3.1-fold increases in total expression (one-sample *t*-test, *p* < 0.05), and 12.2 ± 3.4-fold and 16.9 ± 7.6-fold increases in soluble yield, respectively (Supplementary Figure S1).

For IA, similar patterns were observed (Figure 4c, Table 1). ANOVA indicated significant group effects in both fractions (*p* < 0.0001). Opt-1 and Opt-2 showed marked improvements in total expression (15.3 ± 1.9% and 20.5 ± 2.1% vs. 6.4 ± 2.1%, *p* < 0.01) and soluble fraction (12.6 ± 3.4% and 11.8 ± 1.7% vs. 3.2 ± 0.3%, *p* < 0.01). Fold-change analysis confirmed 2.6–3.4-fold increases in both total and soluble expression for the optimized variants (*p* < 0.05).

Notably, for IA, SCG-GELP optimization not only increased total expression but also substantially improved the soluble protein fraction. IA-Opt1 achieved a soluble fraction of 12.6 ± 3.4%—approximately 3.9-fold higher than WT and nearly double that of the GenScript-optimized sequence (4.2 ± 0.7%). These results demonstrate that SCG-GELP optimizes not only total expression but also proper protein folding and soluble yield, outperforming commercial optimization across independent biological replicates.

### 2.5. DNA Sequence Analysis

To elucidate the molecular mechanisms by which SCG-GELP optimized sequences enhance soluble expression, we performed a codon-by-codon comparison of the wild-type (WT), GenScript-optimized, and SCG-GELP-optimized sequences for both αGP and IA. By comparing the codon compositions of the three sequences, we identified SCG-GELP-specific ‘unique codon’ sites that differed from both the WT and GenScript designs. These unique codons represent the key distinction between our strategy and existing commercial optimization approaches.

**Figure 5:**
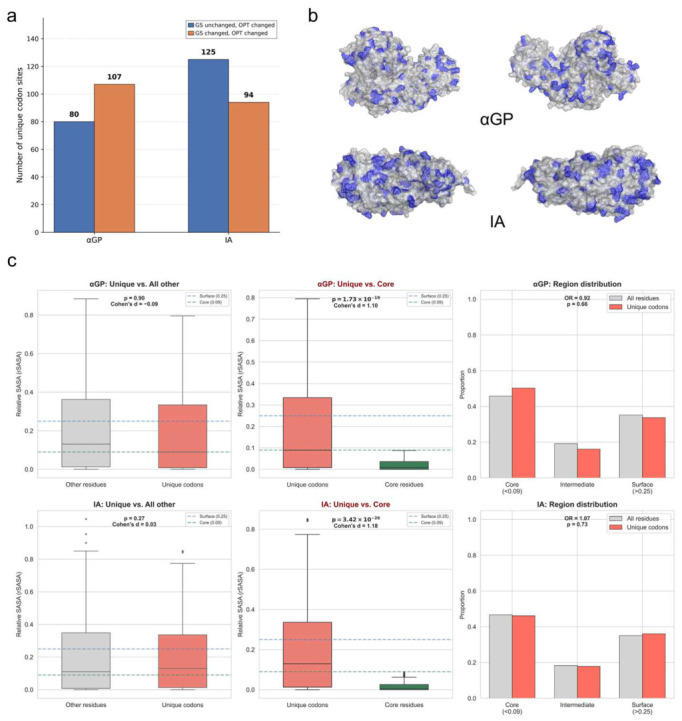
Statistical analysis of unique codon sites and their spatial distribution in protein structures. (**a**) Counts of two classes of unique codon sites. GS unchanged, OPT changed (same): GenScript retained the wild-type (WT) codon, whereas SCG-GELP introduced a different codon, with both optimized sequences identical at this site; GS changed, OPT changed (same): both GenScript and SCG-GELP altered the WT codon but employed different substitutions, with both optimized sequences matching each other. (**b**) Three-dimensional protein structures highlighting amino acid residues corresponding to unique codons (blue). Top row, αGP in two orientations; bottom row, IA in two orientations. (**c**) Quantitative SASA analysis of unique codon sites. Left panels, boxplots comparing rSASA values of unique codons versus all other residues for αGP (**top**) and IA (**bottom**); middle panels, unique codons versus core residues only (rSASA < 0.09); right panels, proportional distribution across core (<0.09), intermediate, and surface (>0.25) regions. Statistical significance was assessed using the Mann–Whitney U test (one-sided) and Fisher’s exact test.

To characterize the distribution pattern of codon changes in SCG-GELP-optimized sequences, we tabulated two classes of “unique codon” sites (Figure 5a; see Supplementary Data). A unique codon was defined as a site simultaneously satisfying three criteria: (1) the codon differs from the wild-type (WT); (2) it differs from the GenScript (GS)-optimized sequence; and (3) both independent SCG-GELP-optimized sequences share the identical codon at this site. These sites were further subdivided based on whether GenScript altered the WT codon. Blue bars denote sites where GenScript retained the WT codon (GS = WT) but SCG-GELP introduced a redesigned codon; orange bars denote sites where both GenScript and SCG-GELP altered the WT codon but employed different substitutions. For αGP, the two categories comprised 80 and 107 sites, respectively; for IA, they comprised 125 and 94 sites, respectively.

Figure 5b illustrates the spatial distribution of amino acid residues corresponding to these unique codons (blue) within the three-dimensional protein structures. The top row shows two orientations of αGP, and the bottom row shows two orientations of IA. These residues were significantly more solvent-exposed than hydrophobic core residues, whereas residues located in the hydrophobic core or within the active pocket were rarely affected, supporting a core-avoiding codon optimization strategy by SCG-GELP. To quantitatively validate this spatial distribution, we calculated the relative solvent accessible surface area (rSASA) for all residues using FreeSASA [34] (Figure 5c). The substituted residues (unique codon sites) exhibited a significantly higher mean rSASA than hydrophobic core residues (rSASA < 0.09) for both αGP (mean 0.187 vs. 0.021; Mann–Whitney U test, *p* = 1.73 × 10^−19^, Cohen’s d = 1.10) and IA (mean 0.205 vs. 0.018; *p* = 3.42 × 10^−26^, Cohen’s d = 1.18).

This preferential avoidance of buried core residues provides an important structural explanation for the experimental results. Traditional global codon optimization strategies, such as GenScript, typically maximize the Codon Adaptation Index (CAI) by distributing codon substitutions uniformly throughout the protein. While this approach can increase total expression, it fails to specifically improve folding efficiency, often leading to higher inclusion body formation. In contrast, the core-avoiding codon adjustments made by SCG-GELP likely improve soluble yields by coordinating translation elongation rates between surface and core regions, thereby optimizing co-translational folding [35]. This is clearly demonstrated in the IA validation: the GenScript-optimized sequence achieved only 4.2 ± 0.7% soluble fraction, whereas IA-Opt1 reached 12.6 ± 3.4%, nearly tripling the functional yield.

## 3. Discussion

The SCG-GELP framework offers an improved approach to codon optimization for recombinant protein expression in *E. coli*. By integrating a Transformer-based synonymous codon generation model with a multi-algorithmic expression prediction system, we have developed a solution that addresses key limitations of existing approaches. Even for a well-studied host such as *E. coli*, traditional CAI-maximizing strategies remain suboptimal because they uniformly distribute high-frequency codons without accounting for the spatial constraints of co-translational folding. Experimental validation with αGP and IA demonstrates the framework’s ability to substantially improve both total protein expression and soluble protein yield, outperforming commercial optimization tools while providing biologically interpretable sequence features.

The substantial improvements in soluble expression observed for both αGP and IA can be attributed to the synergy between large-scale sequence generation and expression-aware screening. While current transformer-based architectures generally perform well in codon optimization, the key advantage of SCG-GELP lies in its ability to generate a massive candidate pool and subsequently prioritize sequences with high soluble expression potential using experimentally validated data. This dual-step strategy effectively couples codon usage compliance with folding-favorable sequence features, enabling the discovery of core-avoiding optimization patterns that are inaccessible to conventional global optimization methods.

A key insight from this study is that SCG-GELP implicitly learns and executes a core-avoiding codon optimization strategy. To our knowledge, this represents the first computationally designed and experimentally validated framework for codon optimization that preferentially avoids buried core residues in industrial enzyme engineering, offering new insights into the complex relationship between DNA sequence and soluble protein expression.

The framework’s success can be attributed to several key factors. First, the use of large-scale, high-quality training data (98,855 *E. coli* sequences for SCG and 2384 expression-annotated sequences for GELP) provides a robust foundation for learning. Second, the integration of multiple algorithmic approaches captures complementary aspects of sequence–expression relationships. Third, the rigorous experimental validation pipeline ensures practical relevance and reliability. These advantages position SCG-GELP as a valuable tool for both basic research and industrial enzyme production.

Several limitations and future directions warrant discussion. Expanding the training data to include more expression-annotated sequences from diverse hosts would enhance the model’s versatility. While effective for single-gene optimization, SCG-GELP currently does not address multi-gene expression balancing—a critical requirement for pathway engineering. Moreover, because the NESG dataset consists primarily of soluble cytosolic and secreted proteins selected for structural genomics, the applicability of SCG-GELP to membrane proteins remains uncertain. Additionally, the NESG dataset’s semi-quantitative expression scores (0–5 scale) may lack the precision of quantitative proteomics, potentially limiting prediction granularity. Although SCG-GELP has demonstrated efficacy in *E. coli*, its generalizability to other expression hosts such as plants, yeast or *Bacillus subtilis* requires further investigation. The experimental validation was performed across three to four independent biological replicates for each protein, with statistical analysis (one-way ANOVA and one-sample *t*-tests) confirming the reproducibility and significance of the observed improvements (Table 1; Figure 4c). For αGP, both optimized variants (Opt-1 and Opt-2) achieved significant increases in total expression (14.5–15.1-fold, *p* < 0.05) and soluble yield (12.2–16.9-fold, *p* < 0.05). For IA, optimized variants showed 2.6–3.4-fold improvements in both total and soluble expression (*p* < 0.05). These results, derived from independent biological replicates rather than a single batch, provide robust quantitative evidence supporting the efficacy of the SCG-GELP framework. Furthermore, while this study experimentally validated the SCG-GELP framework against wild-type sequences and a commercial optimization service (GenScript), a systematic benchmark comparison against other recently developed deep learning-based codon optimization tools was not performed. This is partly because many of these methods lack publicly available pre-trained models or standardized inference pipelines, making fair and reproducible comparisons technically challenging. We contend that for industrial enzyme engineering, the ultimate criterion for codon optimization is the actual soluble protein yield in vivo rather than computational metrics such as CAI or perplexity. In this regard, the experimental results presented here—particularly the substantial improvements over a commercially optimized sequence—provide a biologically meaningful assessment of the framework’s practical utility. Nevertheless, comprehensive benchmarking on standardized datasets remains an important direction for future work.

From an industrial perspective, SCG-GELP offers tangible benefits for enzyme production. Its ability to substantially improve soluble expression—as demonstrated by the 12.2–16.9-fold increase for αGP—directly translates into reduced production costs. Specifically, αGP and IA are critical biocatalysts in our in vitro enzymatic biosystem for industrial myo-inositol production, where their historically low expression levels have constrained scalability. The yield improvements achieved here are expected to significantly lower myo-inositol production costs and to provide robust enzymatic components for expanding the platform to other high-value sugars, including d-Tagatose, glucosamine, and d-Allulose. From a practical standpoint, the framework’s computational efficiency enables rapid sequence optimization. The web interface (https://scg-gelp.biodesign.ac.cn, accessed 3 May 2026) facilitates accessibility for researchers, while open-source availability of the code (https://github.com/yuddecho/SCG-GELP, accessed 3 May 2026) promotes community adoption and further development.

## 4. Materials and Methods

### 4.1. Dataset Preparation

#### 4.1.1. *E. coli* Genomic Dataset RefSeqE

All publicly available protein sequences and corresponding coding DNA sequences of *E. coli* were retrieved from the NCBI Reference Sequence (RefSeq) database and subjected to a comprehensive data cleaning process. The cleaning protocol comprised two principal phases: (1) elimination of sequences exhibiting inconsistencies between protein and DNA sequences based on the standard codon translation table; and (2) implementation of stringent quality criteria, requiring protein sequences to begin with a methionine residue (M), exclude ambiguous residues (X), end with a canonical stop codon (*), and lack internal stop codons (to avoid truncated proteins). Additionally, redundancy removal was performed using MMseqs2 [36,37] with a 30% sequence identity threshold (see Supplementary Methods for detailed commands and parameters). Given the large initial dataset (145 million sequences), batch-wise clustering was followed by merging and deduplication to obtain the final dataset, RefSeqE. We randomly selected 10,000 sequences from RefSeqE as the test set, with the remaining sequences reserved for training (Table 2).

Sequences were encoded using two distinct dictionaries for proteins and DNA. Each dictionary included four reserved tokens (0–3) representing <unk> (unknown character), <pad> (padding character), <bos> (beginning-of-sequence marker), and <eos> (end-of-sequence marker), respectively. The protein dictionary assigned numerical identifiers from 4 to 24 to the 20 canonical amino acids and the stop codon (*), with values inversely correlated to amino acid frequency (lower values indicate higher frequency; see Supplementary Table S1). Similarly, the DNA dictionary assigned identifiers from 4 to 67 to all 64 possible codons, with values determined by their frequencies in the dataset (see Supplementary Table S2).

#### 4.1.2. Gene Expression Level Dataset NESG-DNA

**Figure 6:**
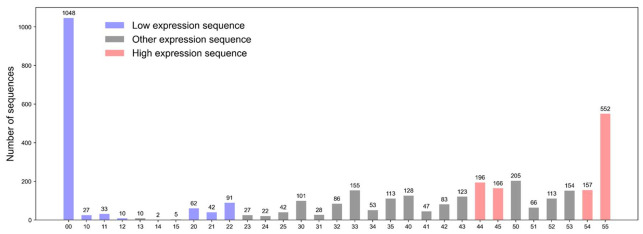
Distribution of gene expression level data. The horizontal coordinate markers indicate a combination of whole-cell fluid and supernatant expression levels, e.g., “54” indicates that the whole-cell fluid protein expression level (E) score is 5 and the supernatant protein expression level (S) score is 4.

Gene expression data were obtained from a large-scale high-throughput protein expression study [38,39,40], encompassing 6348 genes from 2 eukaryotic species, 18 archaeal species, and 151 bacterial species. Proteins were systematically expressed and purified by the Northeast Structural Genomics (NESG) consortium. Whole-cell lysates and supernatants were subjected to SDS-PAGE analysis followed by Coomassie Brilliant Blue staining. Expression levels in whole-cell lysate (E) and supernatant (S) were quantified via visual inspection and assigned integer scores ranging from 0 (no expression) to 5 (maximal expression). Sequences with E and S scores ≤ 2 were classified as low-expression sequences, whereas sequences with E and S scores ≥ 4 were classified as high-expression sequences. As shown in Figure 6, a total of 1313 low-expression (negative) and 1071 high-expression (positive) DNA sequences were obtained to construct the NESG-DNA dataset.

### 4.2. Synonymous Codon Generation (SCG) Model

**Figure 7:**
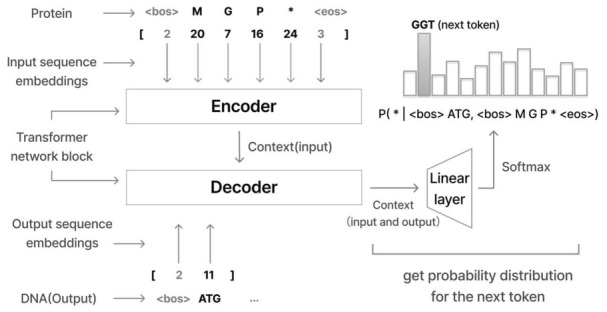
Process of synonymous codon generation using the SCG model. The protein sequence (with * denoting the stop codon) is first transformed via an embedding layer and serves as input to the encoder. The decoder receives both the contextual vectors generated by the encoder and the embedded representations of the partially generated DNA sequence. Through a linear layer followed by a Softmax layer, the model predicts the probability distribution over the next token, thereby generating the output sequence step by step.

The synonymous codon generation (SCG) model adopts a classic encoder–decoder sequence-to-sequence (Seq2Seq) architecture inspired by natural language processing (Figure 7). The model comprises three core components. The first is an embedding layer that transforms sequence tokens into dense vector representations. The second comprises encoder and decoder modules: the encoder analyzes the input protein sequence to extract contextual features, producing a context vector that encapsulates the semantic information of the sequence; the decoder autoregressively generates output sequences based on this context vector and previously generated tokens. The third component is a linear projection layer that transforms decoder outputs into logits (unnormalized probabilities) over the codon vocabulary at each position. Both the encoder and decoder are implemented as Transformer networks [41] with positional encoding. The model hyperparameters are as follows: embedding dimensions of 512 for both encoder (vocabulary size 68) and decoder (vocabulary size 25); 3 Transformer layers; 8 attention heads; hidden layer dimension of 512; Adam optimizer with a learning rate of 0.001; and KLDivLoss (ignoring the <pad> index) as the training objective.

The SCG model utilizes beam search [42] coupled with pruning operations during decoding. At each generation step, the model retains the top *beam_size* candidates ranked by unnormalized probabilities, while immediately discarding codons that would encode mismatched amino acids relative to the target protein sequence. The *beam_size* hyperparameter, which is bounded by the maximum number of synonymous codons for any amino acid (six for leucine), is typically set to 2 or 4 to balance search breadth and computational efficiency. Retained candidates are extended with new codons, and their cumulative log-probabilities are computed. These sequences populate a candidate pool with a predefined capacity (*candidate_pool_size*), which defaults to 1260. The pool is sorted by descending probability, and only the top *candidate_pool_size* entries are retained. Upon decoding completion, the model outputs these top-ranked sequences. Both parameters are user-configurable, ensuring flexibility in optimization granularity and output diversity.

### 4.3. Gene Expression Level Prediction (GELP) Model

**Figure 8:**
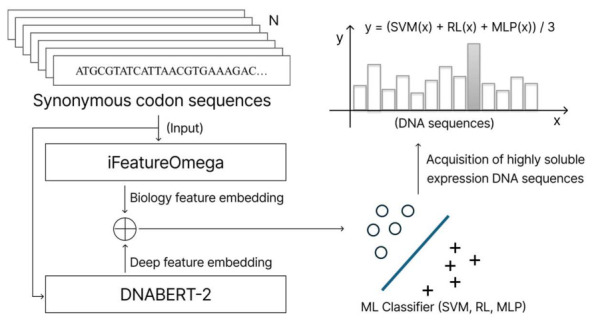
Screening process using the GELP model. The GELP model integrates biological and deep-learning features. The final prediction for identifying DNA sequences with high soluble expression is derived by averaging the independent predictions from a classifier ensemble (SVM, LR, MLP).

The gene expression level prediction (GELP) model comprises two processing steps (Figure 8). First, DNA sequence features are extracted using the DNABERT-2 model. These features are then fed into an ensemble of classifiers—SVM, LR, and MLP—to predict the probability that a given sequence exhibits high soluble expression. The final output is the mean positive-class probability across the three classifiers.

The three classification algorithms, SVM, LR and MLP, were selected from 14 classic machine learning classification algorithms based on the NESG-DNA dataset using ten-fold cross validation (see Supplementary Figure S2 for the performance comparison of all 14 algorithms). We fine-tuned the DNABERT-2 model on a gene expression dataset (NESG-DNA) using default training parameters. The DNABERT-2 contains a Transformer language model consisting of 12 BertEncoder attention layers, with a hidden layer and output size of 768, based on upstream and downstream nucleotide contexts capturing a global and transferable understanding of genomic DNA sequences, and trained on large-scale multi-species genomes.

### 4.4. Experimental Validation

Soluble expression was operationally defined as the target protein yield detected in the soluble fraction after centrifugation of the sonicated cell lysate (8000× *g*, 20 min). Protein levels were quantified by SDS-PAGE and measured as the integrated pixel intensity (absolute band volume) or as the relative band intensity (% of total lane signal). The soluble fraction thus represents proteins that remained in solution under the specified lysis and centrifugation conditions, whereas the pellet fraction contained insoluble aggregates and inclusion bodies. Expression validation was performed across three independent biological replicates for αGP and four for IA. Each replicate was conducted on a separate day with independently prepared cultures. Each DNA sequence was cloned into the pET28a vector (Novagen, Madison, WI, USA) and transformed into *E. coli* BL21 (DE3) cells (New England Biolabs, Ipswich, MA, USA) for protein expression. Single colonies grown on Terrific Broth (TB) agar plates were inoculated into 5 mL of TB liquid medium supplemented with 50 μg mL−1 kanamycin (Sigma-Aldrich, St. Louis, MO, USA). The cultures were incubated at 37 °C with shaking until the optical density at 600 nm (OD600) reached 0.6–0.8. Protein expression was induced by adding isopropyl β-D-1-thiogalactopyranoside (IPTG) (Sigma-Aldrich, St. Louis, MO, USA) to a final concentration of 0.1 mM, followed by incubation at 16 °C for 20–22 h with continuous shaking. Cells were harvested by centrifugation at 6000× *g* for 5 min and resuspended in buffer A (50 mM HEPES, 50 mM NaCl, pH 7.5) (HEPES and NaCl from Sigma-Aldrich, St. Louis, MO, USA). Cell lysis was performed by sonication, and the lysate was clarified by centrifugation at 8000× *g* for 20 min. Protein expression was analyzed by 12% SDS-PAGE for both whole-cell lysates and soluble fractions.

Protein band intensities were quantified using ImageJ Gel Analysis (version 1.54g; National Institutes of Health, Bethesda, MD, USA) [43]. A step-by-step quantification protocol is provided in the Supplementary Methods. For each lane, the absolute band volume (Int) of the target protein band and the total lane signal were recorded. The relative band intensity (%) was calculated as (target band Int/total lane Int) × 100. Fold change was computed as the ratio of the sample’s absolute band volume to that of the corresponding wild-type (WT) control within the same replicate and sample.

Statistical analysis was performed using Python 3.14.5 (Python Software Foundation, Wilmington, DE, USA) with SciPy (version 1.15; SciPy development team, open-source) [44]. For relative band intensity (%), differences among groups were assessed using one-way ANOVA followed by Bonferroni-corrected post hoc *t*-tests (WT vs. each optimized variant; correction factor m = 3). For fold-change data, significance was evaluated using one-sample *t*-tests against a null hypothesis of fold change = 1. Effect sizes are reported as mean ± standard deviation (SD). Significance levels: * *p* < 0.05, ** *p* < 0.01, *** *p* < 0.001; n.s., not significant.

### 4.5. Solvent Accessible Surface Area Calculation

Solvent accessible surface area (SASA) calculation. The AlphaFold (Google DeepMind, London, UK)-predicted structures [18] of αGP (AF-O33831-F1-model_v6) and IA (AF-Q973H3-F1-model_v6) were used for SASA analysis. The relative solvent accessible surface area (rSASA) of each residue was calculated using FreeSASA (v2.2.1; open-source C library, https://freesasa.github.io/, accessed 3 May 2026) with the Lee-Richards algorithm [45] and a solvent probe radius of 1.4 Å. Absolute SASA values were normalised to the maximum accessible surface area of each residue type in an extended Gly-X-Gly tripeptide [46]. Residues with rSASA < 0.09 were classified as buried (core), rSASA > 0.25 as surface-exposed, and intermediate values as partially exposed. Statistical comparisons between unique codon sites and hydrophobic core residues were performed using the Mann–Whitney U test (one-sided). Surface enrichment was assessed using Fisher’s exact test. Effect sizes were reported as Cohen’s d.

## 5. Conclusions

In conclusion, the SCG-GELP framework represents an improved approach to computational protein expression optimization. By combining large-scale sequence learning with multi-algorithmic expression prediction and rigorous experimental validation, we have developed a tool that not only outperforms commercial optimization standards and wild-type sequences but also provides mechanistic insights into the complex relationship between DNA sequence and protein expression. Future work will focus on expanding the framework’s applicability to additional host systems and integrating advanced sequence-structure features to further enhance predictive performance.

## Figures and Tables

**Table 1:** Quantification of αGP and IA expression levels.

Protein	Variant	Sample	Relative Band Intensity (%)	Fold Change (vs. WT)	*p* (vs. WT)
αGP	WT	T	5.2 ± 2.4	1.0	-
		S	4.9 ± 2.9	1.0	-
	GenScript	T	26.6 ± 6.6	8.3 ± 3.5	0.0183 *
		S	27.8 ± 6.8	8.5 ± 1.8	0.0173 *
	Opt-1	T	39.0 ± 0.9	14.5 ± 2.9	0.0001 ***
		S	29.8 ± 1.9	12.2 ± 3.4	0.0007 ***
	Opt-2	T	39.7 ± 1.7	15.1 ± 3.1	0.0001 ***
		S	44.4 ± 4.7	16.9 ± 7.6	0.0007 ***
IA	WT	T	6.4 ± 2.1	1.0	-
		S	3.2 ± 0.3	1.0	-
	GenScript	T	14.6 ± 2.5	2.6 ± 1.3	0.0066 **
		S	4.2 ± 0.7	1.2 ± 0.2	0.1268 n.s.
	Opt-1	T	15.3 ± 1.9	2.6 ± 0.9	0.0023 **
		S	12.6 ± 3.4	3.4 ± 0.9	0.0042 **
	Opt-2	T	20.5 ± 2.1	3.2 ± 0.9	0.0002 ***
		S	11.8 ± 1.7	3.2 ± 0.6	0.0002 ***

Note: SDS-PAGE gels were analyzed using ImageJ Gel Analysis. Relative band intensity (%) indicates the percentage of the target band absolute band volume (Int) relative to the total lane signal. Fold change was calculated by dividing each variant’s absolute band volume by the corresponding wild-type (WT) absolute band volume within the same replicate and sample (intra-replicate normalization). Data represent mean ± standard deviation (SD) of three (αGP) or four (IA) independent biological replicates. Statistical significance for % data: one-way ANOVA followed by Bonferroni-corrected post hoc *t*-tests; for fold change data: one-sample *t*-test (H_0_: fold change = 1). * *p* < 0.05, ** *p* < 0.01, *** *p* < 0.001; n.s., not significant. T (Lysate): whole-cell lysate; S (Supernatant): soluble fraction after centrifugation.

**Table 2:** Changes in the number of sequences in the construction of the RefSeqE dataset.

Step	Number of Sequences
Retrieval of RefSeq data from NCBI	144,775,103
Data cleaning	136,321,237
MMseqs2 30% clustering (RefSeqE dataset)	98,855
Train set: Test set	88,855: 10,000

## Data Availability

The original contributions presented in this study are included in the article/Supplementary Material. Further inquiries can be directed to the corresponding authors.

## Supplementary Materials

The following supporting information can be downloaded at: https://www.mdpi.com/article/10.3390/ijms27115131/s1.
