# Supplementary material for: Deep Learning-Guided Reverse Translation Enhances Soluble Expression of Recombinant Proteins in Escherichia coli
Source: Int J Mol Sci. 2026 Jun 5;27(11):5131. doi: 10.3390/ijms27115131 (PMC13258643; doi:10.3390/ijms27115131)
Supplement: Supplementary file 1 [file ijms-27-05131-s001.zip › Supplementary Material.pdf]

Supplementary Material

Deep Learning-Guided Reverse Translation Enhances Soluble Expression of Recombinant Proteins in *Escherichia coli*

Dong Yu[1], Nan Geng[1], Lin Fan[2,4], Yanmei Qin[3,4], Shangshang Sun[4], Hao Chen[1], Ruoyu Wang[4], Xiaoping Liao[4 *], Chun You[3,4 *]

,,

[1] Tianjin University of Science and Technology, Tianjin 300457, China; yuddecho@gmail.com (D.Y.); gengnans@foxmail.com (N.G.); chenhao24@tib.cas.cn (H.C.)

[2] Sino-Danish College, University of Chinese Academy of Sciences, Beijing, China; fanl@tib.cas.cn (L.F.)

[3] University of Chinese Academy of Sciences, Beijing 100049, China; qinym@tib.cas.cn (Y.Q.)

[4] Tianjin Institute of Industrial Biotechnology, Chinese Academy of Sciences, Tianjin 300308, China; sunshangshang@yeahebio.com (S.S); wangry@tib.cas.cn (R.W.)

[*] Correspondence: yoshion@foxmail.com (C.Y.); liao_xp@tib.cas.cn (X.L.)

Current Address: Chun You: State Key Laboratory of Microbial Metabolism, School of Life Sciences and Biotechnology, Shanghai Jiao Tong University, Shanghai 200240, China; you_c@sjtu.edu.cn (C.Y.)

Supplementary methods

Escherichia coli genome data download and clustering

We used the ncbi-genome-download [1] tool to download the E. coli genome data from NCBI with the following download command: (The tool can be downloaded and used from https://github.com/kblin/ncbi-genome-download)

ncbi-genome-download all --section refseq --genera "Escherichia coli" --assembly-levels all --formats cds-fasta,protein-fasta --output-folder /Volumes/T7 --parallel 64 --retries 16

We used many-against-many sequence searching (MMSeqs) [2] for clustering analysis of gene sequences with the clustering command: (The tool can be downloaded and used from https://github.com/soedinglab/MMseqs2)

mmseqs easy-cluster source_dna_fasta_file nucle_cluster_resule_name temp_dir --min-seq-id 0.9

Sequence assessment indicators (CAI, GC-content and Sequence Identity)

The codon adaptation index (CAI) [3] quantifies a measure of the degree of codon adaptation within a gene by comparing the codon use of the gene to the preferred or optimal codon (which ranges from 0 to 1). CAI was calculated as in (1), where L denotes the sequence length and W_i denotes the ratio of the frequency of use of the i-th codon to the frequency of use of the best codon for the same amino acid (Table S3).

$$CAI=\exp\left(\frac{1}{L}\sum_{i}^{L}\ln W_i\right) \quad (1)$$

GC content indicates the percentage of the gene containing guanine (G) and cytosine (C) nitrogenous bases.

Sequence Identity is often used to describe sequence homology and is calculated as the number of identical residues at the corresponding positions of two gene sequences of the same length as a percentage of the total length.

Classical binary classification machine learning algorithms

A total of 14 machine learning algorithms are involved in our study, all implemented

based on scikit-learn [7], xgboost, and lightgbm libraries. They are support vector machine (SVM) [8], multi-layer perceptron (MLP), logistic regression (LR), adaptive boosting (AdaBoost) [9], latent dirichlet allocation (LDA) [10], extreme gradient boosting (XGBoost) [11], k nearest neighbors (KNN) [12], light gradient boosting machine (LightGBM) [13], decision tree (DT) [14], stochastic gradient descent (SGD), naive bayes (NB) [15], bootstrap aggregation (Bagging) [16], random forest (RF) [17], quadratic discriminant analysis (QDA).

Indicators for evaluating binary classification results

There are four kinds of prediction results for binary classification problems: true positive (TP) is a positive sample that is predicted as positive by the model; false positive (FP) is a negative sample that is predicted as positive by the model; false negative (FN) is a positive sample that is predicted as negative by the model; true negative (TN) is a negative sample that is predicted as negative by the model.

Accuracy (Acc) indicates the percentage of correctly predicted results out of the total sample and is calculated as follows:

$$Acc=\frac{TP+TN}{TP+FP+FN+TN} \tag{2}$$

Precision (Pre) indicates the number of records that are actually positive out of those that are predicted to be positive and is calculated as follows:

$$Pre=\frac{TP}{TP+FP} \tag{3}$$

Recall (Rec) indicates the number of records that are predicted to be positive out of those that are actually positive and is calculated as follows:

$$Rec=\frac{TP}{TP+FN} \tag{4}$$

The F1 score represents the reconciled value of precision and recall and is calculated as follows:

$$\frac{1}{F1}=\frac{1}{Pre}+\frac{1}{Rec} \quad (5)$$

The receiver operating characteristic curve (ROC) is a coordinate graph composed of true positive rate (TPR) as the vertical axis and false positive rate (FPR) as the horizontal axis. Area under the ROC curve (AUC) is calculated by taking the predicted probability values as thresholds one by one, discriminating the prediction results according to the thresholds and calculating the TPR and FPR, which form the ROC curve, and then calculating the area under the ROC curve.The TPR and FPR are calculated as follows:

$$FPR=\frac{FP}{TN+FP} \quad (6)$$

$$FPR=\frac{FP}{TN+FP} \quad (7)$$

Matthews correlation coefficient (MCC) describes the correlation coefficient between the actual classification and the predicted classification in the range [-1, 1] and is calculated as follows:

$$MCC=\frac{TP\times TN-FP\times FN}{\sqrt{(TP+FP)(TP+FN)(TN+FP)(TN+FN)}} \quad (8)$$

ImageJ gel quantification protocol

Protein band intensities were quantified using ImageJ (version 1.54g). The detailed quantification workflow is as follows:

1. **Image import**: Open the raw gel image via File → Open.
2. **Grayscale conversion**: Convert to 8-bit grayscale via Image → Type → 8-bit. Color images must be converted prior to quantification.
3. **Background subtraction**: Perform background correction via Process → Subtract Background. Set the **Rolling ball radius** to 50 pixels, check **Light background**, and click OK.
4. **Image inversion**: Invert the image via Edit → Invert.

5. **Measurement setup**: Configure measurement parameters via Analyze → Set Measurements. Check **Mean gray value** and **Integrated density**, then click OK.
6. **ROI selection and measurement**: Use the **Rectangular Selection Tool** to draw a rectangular region of interest (ROI) around the target protein band. Click Analyze → Measure to record the values. The **RawIntDen** value was used as the absolute band volume. To obtain the total lane signal, a rectangular ROI encompassing the entire lane was drawn and measured in the same manner.

The relative band intensity (%) was calculated as (target band absolute band volume / total lane signal) × 100.

Gene sequences for the experiment

αGP（WT）：

ATGCTGGAGA	AACTTCCCGA	GAACCTGAAA	GAGCTCGAGA	GCCTTGCCTA	CAACCTCTGG	60
TGGAGCTGGT	CCAGACCTGC	TCAGAGACTC	TGGAGAATGA	TCGATTCAGA	AAAGTGGGAG	120
GAACACAGAA	ATCCCGTCAA	AATACTGAGA	GAAGTCTCAA	AGGAAAGACT	GGAAGAACTA	180
TCGAAAGACG	AGGACTTCAT	CGCTCTCTAC	GAACTGACGC	TCGAGAGATT	CACAGACTAC	240
ATGGAAAGGG	AAGACACCTG	GTTCAACGTG	AACTATCCCG	AATGGGACGA	AAAGATAGTT	300
TACATGTGTA	TGGAATACGG	ACTGACGAAA	GCACTTCCGA	TCTACTCTGG	AGGACTCGGT	360
ATCCTTGCCG	GAGACCACCT	CAAATCAGCC	AGTGATCTTG	GCCTTCCTCT	CATAGCCGTA	420
GGTCTTCTTT	ACAAACACGG	GTATTTCACT	CAACAGATAG	ACAGTGACGG	AAGACAGATC	480
GAGATCTTTC	CAGAATACGA	CATCGAAGGA	CTCCCGATGA	AACCTCTCAG	GGATGAAGAC	540
GGAAACCAGG	TGATCGTAGA	AGTACCCATA	GACAACGATA	CTGTAAAAGC	GCGTGTGTTC	600
GAGGTACAGG	TCGGAAGGGT	GAAACTGTAT	CTTCTCGACA	CTGACTTCGA	GGAAAACGAG	660
GATAGATTCA	GAAAGATCTG	CGACTATCTC	TACAATCCCG	AGCCTGATGT	GAGAGTTTCC	720
CAGGAAATTC	TGCTCGGCAT	TGGTGGAATG	AAACTCCTGA	AGACTCTCAA	GATAAAACCT	780
GGAGTCATCC	ACCTGAACGA	AGGTCATCCC	GCTTTTTCAT	CCCTCGAAAG	GATAAAGAGC	840
TACATGGAAG	AAGGATATTC	CTTCACCGAG	GCCCTTGAGA	TCGTCAGACA	GACCACAGTT	900
TTCACGACAC	ACACCCCCGT	CCCCGCAGGT	CACGACAGGT	TCCCGTTCGA	TTTCGTGGAA	960
AAGAAGCTGA	CAAAGTTCTT	CGAAGGATTC	GAATCCAAAG	AACTGCTTAT	GAACCTTGGA	1020
AAAGACGAAG	ACGGAAATTT	CAACATGACG	TATCTTGCTT	TGAGAACCTC	CTCCTTTATA	1080
AACGGAGTGA	GCAAACTTCA	CGCTGACGTA	TCGAGAAGGA	TGTTCAAAAA	TGTCTGGAAG	1140
GGAGTTCCGG	TGGAGGAGAT	CCCCATTGAA	GGCATCACGA	ATGGTGTCCA	CATGGGAACC	1200
TGGATCAACC	GCGAGATGAG	AAAACTGTTC	GACAGGTACC	TCGGTAGAGT	CTGGAGGGAA	1260
CACACTGACC	TCGAAGGAAT	ATGGTACGGA	GTTGACAGAA	TACCCGATGA	AGAACTCTGG	1320
GAAGCGCATC	TGAACGCAAA	GAAACGATTC	ATAGATTACA	TAAGAGAATC	CATCAAAAGG	1380
AGAAACGAAA	GGCTTGGAAT	CAACGAACCA	CTGCCGGAGA	TCAGTGAAAA	CGTGCTCATC	1440
ATAGGTTTTG	CCAGAAGGTT	CGCAACTTAC	AAGAGAGCCG	TCCTGCTCTT	CAGCGATCTG	1500
GAAAGACTCA	AGAGAATTGT	CAATAATTCC	GAGAGGCCGG	TTTACATTGT	GTACGCTGGA	1560

AAGGCCCACC CGAGAGACGA AGGTGGAAAG GAGTTTCTCA GAAGGATCTA CGAAGTTTCA 1620
CAGATGCCCG ATTTCAAGAA CAAAATCATC GTACTCGAAA ACTATGACAT CGGAATGGCT 1680
CGACTCATGG TGTCGGGTGT TGACGTGTGG TTGAACAATC CAAGGAGGCC CATGGAGGCA 1740
AGCGGGACAA GTGGTATGAA AGCTGCAGCG AACGGTGTTC TGAACGCGAG TGTATACGAT 1800
GGCTGGTGGG TTGAGGGATA CAACGGCAGA AACGGATGGG TGATAGGTGA TGAAAGCGTG 1860
CTTCCAGAAA CAGAAGCGGA TGATCCAAAG GATGCCGAGG CTCTGTATGA GCTTCTCGAA 1920
AACGAAATAA TCCCCACCTA CTACGAAAAC AGAGAAAAGT GGATCTTCAT GATGAAAGAA 1980
AGCATAAAAG CGTGGCTCCA AAATTCAGCA CCACCCGCAT GCTGCAAAGA GTACACGGAG 2040
AAATTCTACA TAAAGGGACT TGTGAACAGG GAATGGCTGG AGAGAAGAGA AAACGTCGAA 2100
AAAATCGGAG CCTGGAAAGA AAGAATCCTC AAGAACTGGG AGAATGTTTC CATAGAGCGC 2160
ATTGTTCTTG AAGATTCGAA GAGCGTAGAA GTAACTGTAA AACTGGGCGA TCTCACACCG 2220
AACGACGTGA TAGTCGAACT TGTGGCTGGA AGAGGAGAGG GAATGGAAGA TCTCGAAGTG 2280
TGGAAAGTGA TACACATCAG AAGGTACAGG AAAGAGAACG AATCTATTCG TTTACACTTA 2340
CACCAATGGT GTCTTGGTCA TCTTGGATCT CCCGGATGGT TCTACGCGGT GAGAGTCATA 2400
CCGTACCATC CCAGGCTTCC CATCAAGTTC CTGCCCGAAG TACCGGTTGT CTGGAAGAAG 2460
GTTCTCTGA 2469

αGP（GenScript）：

ATGTTGGAGA AGCTACCCGA AAATTTAAAA GAACTGGAGA GTTTAGCGTA TAACCTGTGG 60
TGGTCATGGA GCCGTCCGGC CCAGCGTCTA TGGCGTATGA TCGACAGCGA AAAGTGGGAA 120
GAACATCGTA ACCCGGTGAA GATTCTGCGT GAGGTAAGCA AAGAGCGCCT GGAGGAACTG 180
TCGAAGGACG AGGACTTCAT TGCGTTGTAC GAATTGACCC TGGAGCGCTT CACTGACTAT 240
ATGGAAAGAG AAGATACCTG GTTCAACGTG AATTACCCGG AGTGGGATGA AAAGATAGTG 300
TATATGTGCA TGGAGTACGG CCTGACGAAA GCCTTGCCGA TCTATAGCGG TGGCCTGGGT 360
ATTTTGGCTG GCGATCATCT GAAAAGCGCT AGCGACCTGG GTTTGCCACT GATTGCGGTT 420
GGCTTGCTGT ATAAACACGG CTACTTCACG CAGCAAATTG ATAGTGACGG ACGCCAAATC 480
GAGATCTTCC CGGAGTACGA CATCGAGGGA CTGCCGATGA AACCGCTGCG TGACGAGGAC 540
GGCAACCAGG TCATCGTGGA GGTTCCGATC GATAATGACA CCGTTAAGGC GCGTGTGTTC 600
GAAGTGCAGG TTGGTCGCGT TAAACTGTAC TTGCTGGATA CTGACTTTGA AGAGAACGAG 660
GACCGATTTC GTAAAATCTG TGATTACCTG TATAACCCGG AACCGGACGT ACGTGTTTCG 720
CAAGAAATTC TGCTGGGTAT CGGTGGTATG AAACTGCTCA AGACCCTGAA GATCAAGCCG 780
GGTGTTATCC ACTTAAACGA AGGCCACCCG GCGTTTTCCA GCCTGGAAAG AATCAAGAGC 840
TATATGGAGG AAGGTTACAG CTTTACCGAA GCTTTGGAGA TCGTGCGTCA AACCACCGTC 900
TTTACGACCC ATACCCCGGT TCCGGCTGGT CACGATCGTT TTCCGTTTGA TTTCGTGGAG 960
AAAAAGTTGA CGAAATTCTT CGAGGGCTTC GAAAGCAAAG AACTGCTTAT GAATCTCGGC 1020
AAGGATGAGG ACGGTAATTT TAACATGACC TATCTAGCGT TGCGTACCAG CAGCTTCATT 1080
AACGGCGTGT CCAAGCTGCA CGCCGATGTC TCTCGTCGTA TGTTCAAAAA CGTGTGGAAA 1140
GGTGTACCGG TAGAGGAAAT TCCGATTGAA GGCATCACCA ATGGTGTTCA CATGGGTACA 1200
TGGATTAACC GTGAGATGCG TAAACTGTTC GATCGCTATT TAGGCCGCGT GTGGCGCGAG 1260
CACACCGATT TGGAGGGGAT CTGGTATGGT GTTGACCGTA TTCCTGATGA GGAACTGTGG 1320
GAAGCCCACC TTAACGCGAA AAAGCGTTTT ATCGACTACA TCCGTGAAAG CATCAAACGT 1380
CGCAACGAAC GCCTAGGCAT CAACGAGCCG CTGCCAGAAA TAAGCGAGAA CGTGCTGATC 1440
ATCGGCTTTG CACGTCGCTT CGCCACCTAC AAGCGCGCAG TTCTGTTATT TTCCGACTTG 1500

GAGCGCCTGA AAAGAATTGT GAACAACAGC GAACGTCCGG TTTACATTGT CTACGCAGGC 1560
AAGGCGCATC CGCGTGATGA GGGTGGTAAA GAATTTCTGA GGCGCATTTA CGAAGTGTCG 1620
CAAATGCCGG ATTTCAAAAA TAAAATTATT GTCTTGGAGA ACTACGACAT CGGTATGGCA 1680
CGTCTCATGG TTTCTGGTGT TGACGTGTGG CTCAACAATC CGCGTCGCCC TATGGAAGCG 1740
TCCGGTACTA GCGGCATGAA GGCGGCAGCC AATGGCGTTC TGAATGCATC CGTGTATGAC 1800
GGTTGGTGGG TTGAAGGCTA CAATGGACGT AACGGCTGGG TTATCGGTGA CGAGTCCGTT 1860
CTGCCGGAGA CAGAAGCTGA TGATCCGAAA GATGCAGAAG CGCTGTACGA GCTGCTGGAG 1920
AACGAGATCA TCCCGACCTA CTACGAAAAC AGGGAGAAGT GGATTTTCAT GATGAAAGAA 1980
TCTATTAAAG CTTGGCTTCA GAATTCAGCG CCACCGGCCT GCTGCAAGGA ATACACCGAA 2040
AAATTCTATA TTAAGGGTCT GGTGAACCGC GAGTGGCTGG AGCGCCGTGA GAATGTTGAA 2100
AAGATCGGTG CGTGGAAGGA GCGTATTTTA AAGAACTGGG AAAACGTTTC TATCGAGCGC 2160
ATCGTTTTGG AGGACAGCAA GTCCGTGGAA GTCACCGTCA AACTGGGTGA CCTCACGCCA 2220
AATGATGTGA TCGTGGAACT GGTTGCAGGT CGTGGTGAAG GGATGGAGGA CTTGGAAGTT 2280
TGGAAAGTTA TTCATATTCG TCGTTATCGT AAAGAGAACG AGTCGATCCG CCTGCATCTG 2340
CACCAGTGGT GTCTGGGCCA CCTGGGCAGC CCGGGTTGGT TTTATGCGGT GCGTGTGATT 2400
CCGTATCATC CGCGTTTGCC CATTAAGTTC CTGCCGGAAG TCCCGGTGGT GTGGAAGAAG 2460
GTTCTGTAA 2469

αGP（Opt-1）：

ATGCTGGAAA AACTGCCGGA AAACCTGAAA GAACTGGAAA GCCTGGCATA CAACCTGTGG 60
TGGAGCTGGA GCCGTCCGGC ACAGCGTCTG TGGCGGATGA TCGACAGCGA AAAATGGGAA 120
GAACATCGTA ACCCGGTGAA AATTCTGCGT GAAGTGTCTA AAGAACGTCT GGAAGAACTG 180
AGCAAAGACG AAGATTTCAT CGCTCTGTAC GAACTGACTC TGGAACGTTT CACCGACTAC 240
ATGGAACGTG AAGATACCTG GTTCAACGTG AACTACCCGG AGTGGGACGA AAAAATCGTT 300
TACATGTGCA TGGAATACGG TCTGACCAAA GCGCTGCCGA TTTACTCTGG TGGTCTGGGT 360
ATCCTGGCGG GCGATCACCT GAAATCTGCG TCTGACCTGG GTCTGCCGCT GATTGCGGTG 420
GGTCTGCTGT ACAAACACGG TTACTTCACT CAGCAGATCG ACTCTGACGG TCGTCAGATC 480
GAAATCTTCC CGGAATACGA TATCGAAGGT CTGCCGATGA AACCGCTGCG TGACGAAGAC 540
GGCAACCAGG TTATCGTTGA AGTTCCGATT GATAACGACA CCGTTAAAGC CCGTGTGTTC 600
GAAGTTCAGG TGGGTCGTGT GAAACTGTAC CTGCTGGACA CCGACTTTGA AGAAAACGAA 660
GATCGTTTCC GTAAAATCTG CGATTACCTG TACAACCCGG AACCGGACGT TCGTGTTTCT 720
CAGGAAATCC TGCTGGGTAT CGGTGGTATG AAACTGCTGA AAACCCTGAA AATCAAACCG 780
GGTGTTATTC ACCTGAACGA AGGTCACCCG GCGTTCTCTT CTCTGGAACG TATCAAATCT 840
TACATGGAAG AAGGTTACTC CTTCACCGAA GCGCTGGAAA TCGTTCGTCA GACCACCGTT 900
TTCACCACTC ACACCCCGGT TCCGGCCGGT CACGACCGTT TCCCGTTTGA CTTCGTTGAG 960
AAAAAACTGA CCAAATTCTT CGAAGGCTTT GAATCTAAAG AACTGCTGAT GAACCTGGGT 1020
AAAGACGAAG ACGGTAACTT TAACATGACC TACCTGGCGC TGCGTACCTC TTCCTTCATC 1080
AACGGTGTTT CTAAACTGCA CGCGGATGTT TCTCGTCGTA TGTTCAAAAA CGTGTGGAAA 1140
GGCGTTCCGG TTGAAGAGAT TCCGATTGAA GGTATCACCA ACGGTGTTCA CATGGGTACC 1200
TGGATCAACC GTGAGATGCG TAAACTGTTC GACCGTTACC TGGGTCGTGT GTGGCGTGAA 1260
CACACCGATC TGGAAGGTAT CTGGTACGGT GTTGACCGTA TTCCGGACGA AGAGCTGTGG 1320
GAAGCGCACC TGAACGCGAA AAAACGTTTC ATTGACTACA TCCGTGAGTC TATCAAACGT 1380
CGTAACGAAC GTCTGGGTAT CAACGAACCG CTGCCGGAAA TCTCTGAAAA CGTGCTGATT 1440

ATCGGTTTTG CGCGTCGTTT TGCGACCTAC AAACGTGCGG TTCTTCTGTT CTCCGACCTG 1500

GAACGTCTGA AACGTATCGT TAACAACAGC GAACGTCCGG TTTACATCGT TTACGCAGGT 1560

AAAGCGCACC CGCGTGACGA AGGTGGTAAA GAGTTCCTGC GTCGTATCTA CGAAGTTTCT 1620

CAGATGCCGG ACTTTAAGAA CAAAATCATC GTTCTGGAAA ACTACGACAT CGGTATGGCC 1680

CGTCTGATGG TTTCCGGTGT TGACGTTTGG CTGAACAACC CGCGCCGTCC GATGGAAGCT 1740

TCCGGTACCT CCGGTATGAA AGCTGCGGCT AACGGTGTTC TGAACGCGTC TGTTTACGAC 1800

GGTTGGTGGG TTGAGGGTTA TAACGGTCGT AACGGTTGGG TTATCGGTGA CGAAAGCGTA 1860

CTGCCGGAAA CCGAAGCGGA CGACCCGAAA GACGCGGAAG CGCTGTACGA ACTGCTGGAA 1920

AACGAGATCA TCCCGACCTA CTACGAAAAC CGTGAAAAAT GGATCTTCAT GATGAAAGAA 1980

TCTATCAAAG CATGGCTGCA GAACAGCGCG CCGCCGGCGT GCTGCAAAGA GTACACCGAG 2040

AAATTCTACA TCAAAGGTCT GGTTAACCGT GAATGGCTGG AACGTCGTGA AAACGTTGAG 2100

AAAATCGGTG CATGGAAAGA GCGTATCCTC AAAAACTGGG AAAACGTTTC TATCGAACGT 2160

ATCGTTCTGG AAGACAGCAA ATCTGTTGAA GTTACCGTTA AACTCGGCGA CCTGACTCCG 2220

AACGACGTTA TCGTTGAACT GGTTGCCGGT CGTGGTGAAG GTATGGAAGA CCTGGAAGTT 2280

TGGAAAGTGA TTCACATCCG TCGTTACCGT AAAGAAAACG AATCCATCCG TCTCCACCTG 2340

CACCAGTGGT GCCTCGGTCA CCTCGGTTCT CCGGGTTGGT TCTACGCGGT TCGTGTTATC 2400

CCGTACCACC CGCGTCTGCC GATCAAATTC CTGCCGGAAG TTCCGGTTGT TTGGAAAAAA 2460

GTTCTGTAA 2469

αGP（Opt-2）：

ATGTTAGAAA AACTACCAGA AAATTTAAAA GAGCTGGAAT CACTGGCTTA TAACCTGTGG 60

TGGTCATGGA GCCGTCCGGC TCAGCGTTTA TGGCGTATGA TTGACTCTGA AAAATGGGAA 120

GAACACCGTA ATCCGGTAAA AATTCTGCGT GAAGTCAGCA AAGAACGTCT GGAAGAACTG 180

AGCAAAGACG AAGATTTTAT CGCGCTGTAC GAACTGACGC TGGAACGTTT CACCGACTAC 240

ATGGAACGTG AAGACACCTG GTTCAACGTT AACTACCCGG AATGGGACGA AAAAATCGTT 300

TACATGTGCA TGGAATACGG TCTGACCAAA GCGCTGCCGA TTTACAGCGG TGGTCTGGGT 360

ATTCTGGCTG GCGATCACCT GAAATCTGCT TCCGACCTGG GTCTGCCGCT GATTGCCGTA 420

GGTCTGCTGT ACAAACACGG TTACTTCACC CAGCAGATCG ACTCTGACGG TCGTCAGATC 480

GAAATCTTCC CGGAATACGA TATCGAAGGT CTGCCGATGA AACCGCTGCG TGATGAAGAC 540

GGTAACCAGG TTATCGTTGA AGTGCCGATC GACAACGACA CCGTGAAAGC GCGTGTGTTC 600

GAAGTTCAGG TTGGTCGCGT GAAACTGTAC CTGCTGGATA CCGACTTCGA AGAAAACGAA 660

GATCGTTTCC GTAAAATCTG CGACTACCTC TACAACCCGG AACCGGACGT GCGTGTTTCT 720

CAGGAAATCC TGCTGGGTAT CGGTGGTATG AAACTGCTGA AAACCCTGAA AATCAAACCG 780

GGTGTGATTC ACCTGAACGA AGGTCACCCG GCGTTCTCTT CTCTGGAACG TATCAAATCT 840

TACATGGAAG AAGGTTACTC TTTCACCGAA GCGCTGGAAA TCGTTCGTCA GACCACCGTG 900

TTCACCACTC ACACTCCGGT TCCGGCAGGT CACGACCGTT TCCCGTTCGA CTTCGTTGAG 960

AAAAAACTGA CCAAATTCTT CGAAGGCTTC GAATCTAAAG AACTGCTGAT GAACCTCGGT 1020

AAAGATGAAG ACGGTAACTT CAACATGACC TATCTGGCGC TGCGTACCTC TTCCTTCATC 1080

AACGGTGTTT CTAAACTGCA CGCGGACGTT TCTCGTCGTA TGTTCAAAAA CGTCTGGAAA 1140

GGCGTTCCGG TTGAAGAGAT TCCGATCGAA GGTATCACCA ACGGTGTTCA CATGGGTACC 1200

TGGATCAACC GTGAGATGCG TAAACTGTTT GACCGTTACC TGGGTCGTGT TTGGCGTGAA 1260

CACACCGACC TGGAAGGTAT CTGGTACGGT GTTGACCGTA TTCCGGACGA AGAGCTGTGG 1320

GAAGCGCACC TGAACGCGAA GAAACGTTTC ATCGACTACA TCCGTGAGTC TATCAAACGT 1380

CGTAACGAAC GTCTGGGTAT CAACGAACCG CTGCCGGAAA TCTCTGAAAA CGTGCTGATT 1440
ATCGGTTTTG CGCGTCGTTT TGCGACCTAC AAACGTGCGG TTCTGCTGTT CTCTGACCTG 1500
GAGCGTCTGA AACGTATCGT TAACAACAGC GAACGTCCGG TTTACATCGT TTACGCGGGT 1560
AAAGCGCACC CGCGTGACGA AGGCGGTAAA GAATTCCTGC GTCGTATCTA CGAAGTTTCT 1620
CAGATGCCGG ACTTCAAGAA CAAAATCATC GTTCTGGAAA ACTACGACAT CGGTATGGCG 1680
CGTCTGATGG TTTCTGGTGT TGACGTTTGG CTGAACAACC CGCGTCGTCC GATGGAAGCG 1740
TCCGGTACCA GCGGTATGAA AGCAGCGGCT AACGGTGTTC TGAACGCGTC TGTTTACGAC 1800
GGTTGGTGGG TTGAAGGTTA TAACGGTCGT AACGGTTGGG TTATCGGTGA CGAGTCTGTT 1860
CTGCCGGAAA CCGAAGCGGA TGACCCGAAA GACGCAGAAG CGCTGTACGA ACTGCTGGAA 1920
AACGAGATCA TCCCGACCTA CTACGAAAAC CGTGAAAAAT GGATCTTCAT GATGAAAGAA 1980
TCTATCAAAG CATGGCTGCA GAACAGCGCG CCGCCGGCGT GCTGCAAAGA GTACACCGAG 2040
AAATTCTACA TCAAAGGTCT GGTTAACCGT GAATGGCTGG AACGTCGTGA AAACGTTGAG 2100
AAGATCGGTG CATGGAAAGA GCGTATCCTG AAAAACTGGG AAAACGTTTC TATCGAGCGT 2160
ATCGTTCTGG AAGACAGCAA ATCTGTTGAA GTTACCGTTA AACTCGGCGA CCTGACTCCG 2220
AACGACGTTA TCGTTGAACT GGTTGCGGGT CGTGGTGAAG GTATGGAAGA CCTGGAAGTT 2280
TGGAAAGTTA TCCACATCCG TCGTTACCGT AAAGAAAACG AATCCATCCG TCTGCACCTG 2340
CACCAGTGGT GCCTGGGTCA CCTCGGTTCT CCGGGTTGGT TCTACGCGGT TCGTGTTATC 2400
CCGTACCACC CGCGTCTGCC GATCAAATTC CTGCCGGAAG TTCCGGTTGT TTGGAAAAAA 2460
GTTCTGTAA 2469

IA（WT）：

ATGGTTTTTT CACACAAGGA TAGACCATTA AGACCAGGAG AGCCATATCC TCTTGGAGCT 60
AATTGGGAAG AAGAAGATGA TGGTGTGAAC TTCTCTATCT TTTCGGAAAA TGCGACTAAG 120
GTTGAACTTT TAATTTACTC CCCTACTAAT CAGAAATATC CTAAAGAAGT TATCGAGGTT 180
AAGCAGAGAT CTGGTGATAT TTGGCACGTC TTTGTCCCAG GTTTGGGACC GGGTACACTT 240
TACGCATATA GAATTTATGG TCCTTATAAG CCAGATCAAG GTTTAAGATT TAATCCTAAT 300
AAGGTTCTAA TTGATCCTTA TGCTAAGGCT ATAAATGGGA CATTAAACTG GAATGATGCT 360
GTTTTTGGTT ATAAGATAGG CGATTCTAAC CAGGATTTGT CCTTTGATGA TAGGCCAGAT 420
GATGAATTTA TTCCTAAGGG TGTTGTTATT AATCCTTATT TTGAGTGGGA TGATGATCAC 480
TTTTTTAGGA GAAAGAAGAT ACCATTAAAG GATACTATTA TTTATGAAGT TCATGTTAAA 540
GGTTTTACTA AATTAAGACC TGATTTACCA GAAAATATTA GAGGTACTTA TAAAGGATTT 600
GCCTCTAGAC AGATGATCGA ATATTTGAAA GATTTGGGGG TAACTACAGT CGAGATAATG 660
CCAGTACAGC AGTTTGTTGA TGATAGGTTT CTAGTAGAGA AGGGATTAAG GAATTACTGG 720
GGATATAATC CCATAAATTA TTTTTCACCT GAATGTAGAT ATTCCTCTTC TGGCTGTATG 780
GGTGAACAAG TTAACGAGTT TAAGGAGATG GTTAATGAGC TGCACAACGC TGGCTTCGAG 840
GTAATTATTG ATGTTGTTTA TAACCATACT GCGGAAGGGA ATCATTTAGG TCCTACTCTT 900
TCATTCAGAG GTATAGATAA TTTGGCTTAT TACATGTTAG TTCCAGATAA TAAGAGATAT 960
TATTTAGACT TTACTGGAAC TGGAAACACC TTAAATCTGA GTCATCCGAG GGTATTGCAA 1020
ATGGTTCTGG ATAGTCTTAG ATATTGGGTT TTAGAGATGC ATGTTGACGG TTTTAGGTTT 1080
GATTTAGCTG CTGCCCTAGC TAGACAATTA TACAGTGTAA ATATGCTTTC AACTTTCTTT 1140
GTTGCAATTC AGCAAGATCC CGTTCTTTCT CAAGTTAAGT TAATAGCGGA ACCTTGGGAT 1200
GTTGGTCCAG GGGGATATCA GGTTGGTAAT TTTCCATATT TGTGGGCCGA ATGGAACGGT 1260
AAGTATAGAG ATACTATAAG GAGATTTTGG AGAGGTGAGG CGATCCCCTA TGAGGAGTTG 1320

GCTAATAGGC TTATGGGTTC TCCAGATTTA TATGCTGGAA ATAATAAGAC TCCTTTCGCT 1380
AGTATAAATT ATATAACTTC TCATGATGGT TTTACTTTAG AGGATTTAGT TAGTTATAAT 1440
CAAAAGCATA ATGAAGCTAA CGGTTTTAAT AATCAAGATG GCATGAACGA GAATTATAGT 1500
TGGAATTGTG GAGTTGAGGG AGAGACTAAT GATGCTAATG TTATTCAATG TAGAGAGAAA 1560
CAAAAAAGGA ATTTTATCAT AACACTTTTT GTAAGTCAAG GGGTTCCAAT GATTTTAGGT 1620
GGCGATGAGC TAAGTAGAAC ACAAAGAGGA AATAACAATG CTTTTTGCCA AGATAACGAA 1680
ATAAGTTGGT TTAATTGGAA TCTTGATGAG AGGAAACAGA GGTTTCATGA TTTTGTTAGG 1740
AGTATGATTT ATTTCTATAG AGCTCATCCA ATATTTAGAA GAGAAAGATA CTTTCAAGGT 1800
AAGAAATTAC ATGGTATGCC ATTAAAGGAT GTCACTTTTC TAAAACCAGA TGGAAATGAA 1860
GCTGACGAAC AAACATGGAA GTCACCAACT AATTTTATTG CATATATTTT AGAGGGTAGT 1920
GTTATTGATG AAGTAAATGA TAGGGGTGAG AGAATAGCTG ACGATTCTTT CTTAATCATC 1980
CTTAATGGTT CACCAAATAA TATTAAGTTC AAATTCCCGC AAGGTAAATG GAGTTTAGTT 2040
GTTTCTTCAT ATTTGAGAGA ACTTAGAGAT GACGAGAGAG TTGTTGATGG TGGCAAGGAA 2100
CTGGAAATTG AGGGAAGGAC CGCAATGGTA TATAGGAGGA TTGAATATTA G 2151

IA（GenScript）：

ATGGTATTTT CACACAAAGA TAGGCCCCTA CGTCCTGGTG AACCGTACCC ACTGGGTGCA 60
AACTGGGAAG AAGAGGACGA CGGCGTAAAT TTCTCGATCT TTAGCGAAAA TGCGACCAAA 120
GTGGAGCTAC TGATTTACAG CCCGACGAAC CAAAAGTATC CGAAGGAAGT TATCGAAGTT 180
AAACAACGTA GCGGTGATAT TTGGCATGTG TTTGTGCCCG GCCTGGGTCC AGGGACTCTG 240
TATGCCTACC GTATATATGG TCCGTATAAA CCGGATCAGG GTCTGCGCTT CAACCCGAAT 300
AAAGTCTTGA TCGATCCGTA TGCGAAAGCG ATTAACGGGA CCTTGAATTG GAACGATGCT 360
GTGTTTGGTT ATAAGATCGG CGACTCAAAT CAGGACTTGA GCTTTGATGA CCGTCCCGAC 420
GACGAGTTCA TCCCGAAAGG TGTGGTGATC AATCCGTACT TCGAGTGGGA TGACGACCAC 480
TTTTTCAGAC GCAAAAAAAT TCCACTGAAG GATACCATCA TCTACGAGGT TCATGTCAAA 540
GGCTTCACGA AGCTGCGTCC GGATCTGCCG GAAAACATCC GCGGTACCTA TAAGGGTTTC 600
GCTAGCCGTC AAATGATTGA GTACTTGAAG GACCTCGGCG TTACTACCGT TGAGATCATG 660
CCGGTTCAGC AGTTTGTCGA CGATCGTTTC TTGGTAGAGA AGGGGCTGCG CAACTATTGG 720
GGTTATAACC CGATTAATTA CTTTTCTCCG GAGTGCCGCT ACAGCTCCTC CGGCTGCATG 780
GGTGAACAGG TTAATGAATT CAAAGAGATG GTGAATGAAT TACACAACGC TGGTTTTGAA 840
GTGATTATTG ATGTGGTCTA CAACCACACC GCGGAAGGCA ACCATCTGGG TCCGACGCTG 900
TCGTTCCGTG GTATTGACAA TCTGGCTTAC TATATGTTAG TCCCGGATAA CAAACGTTAT 960
TACCTGGACT TTACCGGTAC TGGTAACACC TTGAACCTGA GCCACCCGCG TGTTCTGCAG 1020
ATGGTTCTGG ACAGCCTCAG ATACTGGGTT CTGGAGATGC ATGTTGACGG TTTCCGCTTT 1080
GACCTGGCGG CAGCGCTAGC TCGTCAACTG TATTCCGTGA ACATGCTTTC GACCTTTTTT 1140
GTTGCAATTC AACAAGACCC GGTCCTGTCG CAAGTTAAGC TGATTGCCGA GCCGTGGGAT 1200
GTTGGTCCGG GTGGTTATCA GGTGGGCAAC TTCCCGTACC TCTGGGCAGA ATGGAACGGC 1260
AAGTACCGGG ATACGATTCG CCGCTTCTGG CGTGGCGAGG CCATCCCGTA CGAAGAACTG 1320
GCGAACCGTC TGATGGGCTC TCCGGATTTG TACGCGGGTA ACAACAAGAC CCCGTTTGCG 1380
TCTATTAACT ACATCACCAG CCACGACGGC TTCACCCTGG AGGATTTAGT GAGCTACAAC 1440
CAGAAGCACA ATGAAGCGAA CGGCTTCAAC AACCAAGACG GTATGAATGA GAATTACTCC 1500
TGGAACTGCG GTGTTGAAGG CGAAACCAAT GACGCCAACG TGATCCAGTG TCGTGAAAAA 1560
CAAAAGCGCA ATTTTATCAT CACCTTGTTT GTTTCCCAGG GCGTCCCGAT GATCCTGGGC 1620

GGCGATGAAC	TGAGCCGTAC	GCAGCGTGGC	AACAACAATG	CGTTTTGTCA	GGACAATGAA	1680
ATCAGCTGGT	TTAACTGGAA	TCTGGACGAG	CGTAAACAGC	GTTTCCACGA	TTTCGTGCGT	1740
TCCATGATTT	ACTTCTATCG	TGCACACCCG	ATCTTCCGTC	GCGAGCGCTA	CTTCCAGGGC	1800
AAAAAGCTGC	ATGGCATGCC	GTTGAAGGAC	GTGACGTTTC	TGAAGCCGGA	TGGTAACGAG	1860
GCTGATGAGC	AAACCTGGAA	AAGCCCGACC	AATTTCATCG	CGTATATTCT	GGAGGGCAGC	1920
GTGATCGACG	AGGTTAACGA	CCGTGGTGAA	CGTATTGCGG	ATGATTCTTT	TCTGATCATT	1980
TTGAACGGTT	CTCCAAATAA	TATTAAATTC	AAATTTCCGC	AGGGCAAGTG	GAGCCTGGTG	2040
GTGTCCAGCT	ATTTAAGAGA	GTTGCGCGAT	GATGAGCGCG	TTGTGGATGG	TGGCAAGGAA	2100
CTGGAGATCG	AGGGTCGTAC	CGCAATGGTT	TACCGTCGCA	TTGAATATTA	A 2151	

IA（Opt-1）：

ATGGTATTTA	GCCACAAAGA	TCGCCCGCTG	CGTCCGGGTG	AACCGTACCC	GCTGGGTGCG	60
AACTGGGAAG	AAGAAGACGA	CGGTGTTAAC	TTCTCTATCT	TCTCTGAAAA	CGCGACTAAA	120
GTTGAACTGC	TGATCTACTC	TCCGACCAAC	CAGAAATATC	CGAAAGAAGT	TATCGAAGTG	180
AAACAGCGTT	CCGGTGATAT	CTGGCACGTC	TTCGTTCCGG	GTCTGGGTCC	GGGTACTCTG	240
TACGCGTACC	GTATCTACGG	CCCGTACAAA	CCGGATCAGG	GTCTGCGTTT	TAACCCGAAC	300
AAAGTTCTGA	TCGACCCGTA	TGCGAAAGCG	ATTAACGGTA	CCCTGAACTG	GAACGACGCG	360
GTTTTCGGTT	ACAAAATCGG	TGACTCCAAC	CAGGACCTGT	CTTTTGACGA	CCGTCCGGAC	420
GACGAATTCA	TCCCGAAAGG	TGTTGTTATC	AACCCGTACT	TTGAATGGGA	CGACGACCAC	480
TTCTTCCGCC	GTAAGAAAAT	CCCGCTGAAA	GACACCATCA	TCTACGAAGT	TCACGTTAAA	540
GGTTTCACCA	AACTGCGTCC	AGACCTGCCG	GAAAACATCC	GTGGCACCTA	CAAAGGTTTC	600
GCGTCTCGTC	AGATGATCGA	ATACCTGAAA	GACCTGGGCG	TTACCACCGT	TGAAATCATG	660
CCAGTTCAGC	AGTTCGTAGA	CGATCGTTTC	CTGGTTGAGA	AAGGTCTGCG	TAACTACTGG	720
GGTTACAACC	CGATCAACTA	CTTCTCTCCG	GAATGCCGTT	ACTCCTCTTC	TGGCTGCATG	780
GGTGAGCAGG	TTAACGAATT	CAAAGAGATG	GTTAACGAAC	TGCACAACGC	GGGTTTCGAA	840
GTGATCATCG	ACGTGGTTTA	CAACCACACC	GCGGAAGGTA	ACCACCTCGG	TCCGACCCTG	900
AGCTTCCGTG	GTATCGACAA	CCTGGCGTAC	TACATGCTGG	TTCCGGACAA	CAAACGTTAC	960
TACCTCGACT	TCACCGGTAC	CGGTAACACC	CTGAACCTGT	CTCACCCGCG	CGTTCTGCAG	1020
ATGGTTCTGG	ACTCTCTGCG	TTACTGGGTT	CTGGAAATGC	ACGTTGACGG	CTTCCGCTTC	1080
GACCTGGCGG	CGGCGCTGGC	GCGTCAGCTG	TACTCTGTGA	ACATGCTGTC	TACCTTCTTC	1140
GTTGCGATCC	AGCAAGACCC	GGTTCTGTCT	CAGGTGAAAC	TGATCGCGGA	ACCGTGGGAC	1200
GTTGGTCCGG	GCGGTTACCA	GGTTGGTAAC	TTCCCGTACC	TGTGGGCGGA	ATGGAACGGC	1260
AAATACCGTG	ACACCATCCG	TCGTTTCTGG	CGTGGTGAAG	CGATCCCGTA	CGAAGAACTG	1320
GCGAACCGTC	TGATGGGTTC	TCCGGACCTG	TACGCGGGTA	ACAACAAAAC	CCCGTTTGCG	1380
TCTATCAACT	ACATCACCTC	TCACGACGGC	TTCACCCTGG	AAGACCTGGT	TTCTTACAAC	1440
CAGAAACACA	ACGAAGCGAA	CGGCTTCAAC	AACCAGGACG	GCATGAACGA	AAACTACTCC	1500
TGGAACTGCG	GTGTTGAAGG	TGAAACCAAC	GACGCGAACG	TTATCCAGTG	CCGTGAGAAA	1560
CAGAAACGTA	ACTTCATCAT	CACCCTGTTC	GTTTCTCAGG	GTGTTCCGAT	GATCCTCGGC	1620
GGTGACGAAC	TGTCTCGTAC	CCAGCGTGGT	AACAACAACG	CGTTCTGCCA	GGACAACGAA	1680
ATCTCCTGGT	TCAACTGGAA	CCTGGACGAA	CGTAAACAGC	GTTTCCACGA	CTTCGTTCGC	1740
AGCATGATCT	ACTTCTACCG	TGCGCACCCG	ATCTTCCGTC	GTGAACGTTA	CTTCCAGGGT	1800
AAAAAACTGC	ATGGTATGCC	GCTGAAAGAC	GTTACCTTCC	TGAAACCGGA	CGGTAACGAA	1860
GCGGATGAAC	AGACCTGGAA	ATCTCCGACC	AACTTCATCG	CTTACATCCT	GGAAGGTTCT	1920

GTTATCGACG AAGTGAACGA CCGTGGTGAA CGTATCGCGG ATGACTCTTT CCTGATCATC 1980
CTGAACGGTT CTCCGAACAA CATCAAATTC AAATTCCCGC AGGGTAAATG GTCTCTGGTT 2040
GTTTCTTCTT ACCTGCGTGA ACTGCGTGAT GACGAACGTG TTGTTGACGG TGGTAAAGAA 2100
CTGGAAATCG AAGGTCGTAC CGCGATGGTT TACCGTCGTA TCGAATACTA A 2151

IA（Opt-2）：

ATGGTATTTA GCCACAAAGA TCGCCCGCTG CGTCCGGGTG AACCGTACCC GCTGGGTGCG 60
AACTGGGAAG AAGAAGACGA CGGTGTTAAC TTCTCTATCT TCTCTGAAAA CGCGACTAAA 120
GTTGAACTGC TGATCTACTC TCCGACCAAC CAGAAATATC CGAAAGAAGT TATCGAAGTG 180
AAACAGCGTT CCGGTGATAT CTGGCACGTC TTCGTTCCGG GTCTGGGTCC GGGTACTCTG 240
TACGCGTACC GTATCTACGG CCCGTACAAA CCGGATCAGG GTCTGCGTTT TAACCCGAAC 300
AAAGTTCTGA TCGACCCGTA TGCGAAAGCG ATTAACGGTA CCCTGAACTG GAACGACGCG 360
GTTTTCGGTT ACAAAATCGG TGACTCCAAC CAGGACCTGT CTTTTGACGA CCGTCCGGAC 420
GACGAATTCA TCCCGAAAGG TGTTGTTATC AACCCGTACT TTGAATGGGA CGACGACCAC 480
TTCTTCCGCC GTAAGAAAAT CCCGCTGAAA GACACCATCA TCTACGAAGT TCACGTTAAA 540
GGTTTCACCA AACTGCGTCC AGACCTGCCG GAAAACATCC GTGGCACCTA CAAAGGTTTC 600
GCGTCTCGTC AGATGATCGA ATACCTGAAA GACCTGGGCG TTACCACCGT TGAAATCATG 660
CCAGTTCAGC AGTTCGTAGA CGATCGTTTC CTGGTTGAGA AAGGTCTGCG TAACTACTGG 720
GGTTACAACC CGATCAACTA CTTCTCTCCG GAATGCCGTT ACTCCTCTTC TGGCTGCATG 780
GGTGAGCAGG TTAACGAATT CAAAGAGATG GTTAACGAAC TGCACAACGC GGGTTTCGAA 840
GTGATCATCG ACGTGGTTTA CAACCACACC GCGGAAGGTA ACCACCTCGG TCCGACCCTG 900
AGCTTCCGTG GTATCGACAA CCTGGCGTAC TACATGCTGG TTCCGGACAA CAAACGTTAC 960
TACCTCGACT TCACCGGTAC CGGTAACACC CTGAACCTGT CTCACCCGCG CGTTCTGCAG 1020
ATGGTTCTGG ACTCTCTGCG TTACTGGGTT CTGGAAATGC ACGTTGACGG CTTCCGCTTC 1080
GACCTGGCGG CGGCGCTGGC GCGTCAGCTG TACTCTGTGA ACATGCTGTC TACCTTCTTC 1140
GTTGCGATCC AGCAAGACCC GGTTCTGTCT CAGGTGAAAC TGATCGCGGA ACCGTGGGAC 1200
GTTGGTCCGG GCGGTTACCA GGTTGGTAAC TTCCCGTACC TGTGGGCGGA ATGGAACGGC 1260
AAATACCGTG ACACCATCCG TCGTTTCTGG CGTGGTGAAG CGATCCCGTA CGAAGAACTG 1320
GCGAACCGTC TGATGGGTTC TCCGGACCTG TACGCGGGTA ACAACAAAAC CCCGTTTGCG 1380
TCTATCAACT ACATCACCTC TCACGACGGC TTCACCCTGG AAGACCTGGT TTCTTACAAC 1440
CAGAAACACA ACGAAGCGAA CGGCTTCAAC AACCAGGACG GCATGAACGA AAACTACTCC 1500
TGGAACTGCG GTGTTGAAGG TGAAACCAAC GACGCGAACG TTATCCAGTG CCGTGAGAAA 1560
CAGAAACGTA ACTTCATCAT CACCCTGTTC GTTTCTCAGG GTGTTCCGAT GATCCTCGGC 1620
GGTGACGAAC TGTCTCGTAC CCAGCGTGGT AACAACAACG CGTTCTGCCA GGACAACGAA 1680
ATCTCCTGGT TCAACTGGAA CCTGGACGAA CGTAAACAGC GTTTCCACGA CTTCGTTCGC 1740
AGCATGATCT ACTTCTACCG TGCGCACCCG ATCTTCCGTC GTGAACGTTA CTTCCAGGGT 1800
AAAAAACTGC ATGGTATGCC GCTGAAAGAC GTTACCTTCC TGAAACCGGA CGGTAACGAA 1860
GCGGATGAAC AGACCTGGAA ATCTCCGACC AACTTCATCG CTTACATCCT GGAAGGTTCT 1920
GTTATCGACG AAGTGAACGA CCGTGGTGAA CGTATCGCGG ATGACTCTTT CCTGATCATC 1980
CTGAACGGTT CTCCGAACAA CATCAAATTC AAATTCCCGC AGGGTAAATG GTCTCTGGTT 2040
GTTTCTTCTT ACCTGCGTGA ACTGCGTGAC GACGAACGTG TTGTTGACGG CGGTAAAGAA 2100
CTGGAAATCG AAGGTCGTAC TGCGATGGTT TACCGTCGTA TCGAATACTA A 2151

Supplementary figures

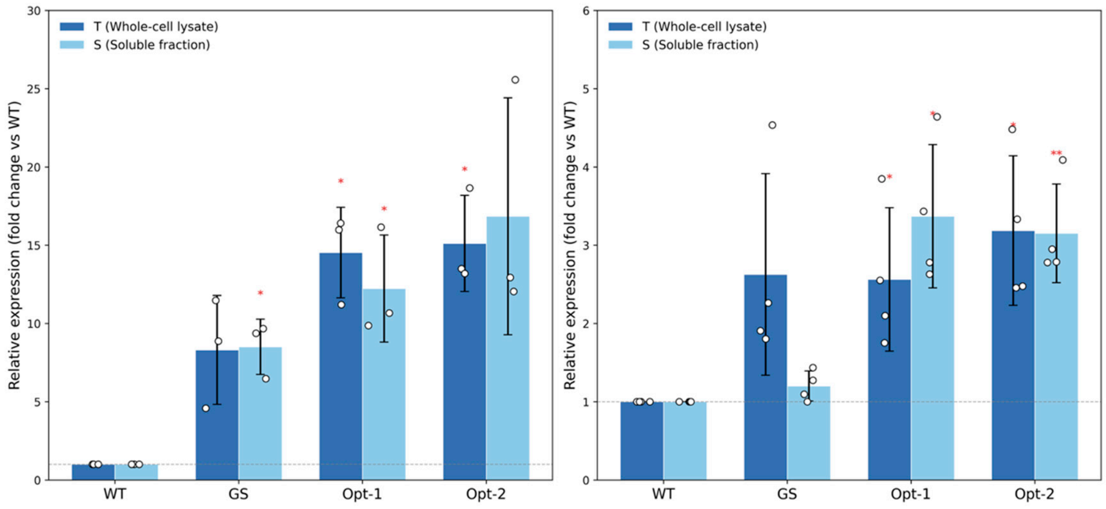

Figure S1. Fold-change analysis of absolute band volume across independent biological replicates. Fold change was calculated as the ratio of the sample's absolute band volume to that of the corresponding WT control within each replicate. (Left) αGP (n = 3). (Right) IA (n = 4). Bars represent mean ± SD. Open circles denote individual replicate values. Statistical significance: one-sample t-test (H_0: fold change = 1). *$p < 0.05$, **$p < 0.01$; n.s., not significant.

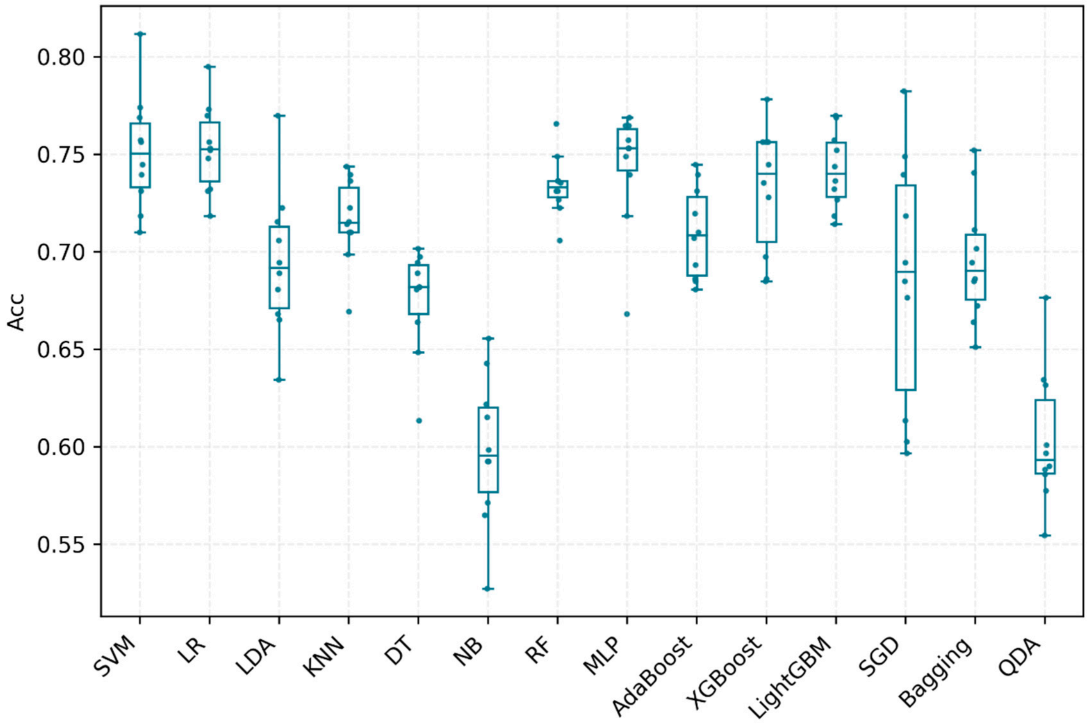

Figure S2. Performance comparison of 14 machine learning algorithms based on

DNABERT-2 embeddings. The accuracy (ACC) of each algorithm was evaluated using 10-fold cross-validation and is presented as box plots overlaid with individual data points. The top three performing algorithms with the highest overall ACC distributions are Logistic Regression (LR), Support Vector Machine (SVM), and Multilayer Perceptron (MLP).

Supplementary tables

Table S1 Amino acid and special symbol coding dictionary

Symbol	Count	Identifier	Symbol	Count	Identifier
<unk>		0	D	1872430	13
<pad>		1	R	1806143	14
<bos>		2	N	1662266	15
<eos>		3	P	1375984	16
L	3187330	4	F	1364149	17
A	2888056	5	Q	1352339	18
S	2357504	6	Y	1156866	19
G	2343504	7	M	857119	20
I	2186251	8	H	679054	21
V	2186167	9	W	487768	22
E	2033673	10	C	411891	23
T	1964981	11	*	145663	24
K	1941951	12			

<unk> represents an unknown character, <pad> represents a padding character, <bos> represents a begin character, and <eos> represents an end character.

Table S2 Nucleotide and special symbol coding dictionary

Symbol	Count	Identifier	Symbol	Count	Identifier
<unk>		0	CGT	483648	34
<pad>		1	CGC	481649	35
<bos>		2	TCA	481445	36
<eos>		3	AGC	476218	37
AAA	1346547	4	CCG	474803	38
GAA	1291818	5	ATA	473285	39
GAT	1232914	6	GGA	462569	40
CTG	1085923	7	GTA	450605	41
ATT	1037544	8	ACT	442280	42
AAT	947429	9	TCT	432016	43
ATG	857119	10	ACG	429860	44
TTT	853528	11	TAC	429853	45
CAG	837575	12	TTG	427077	46
GCA	799812	13	GGG	424868	47
GGT	775631	14	AGT	424116	48
GAG	741855	15	CAT	417481	49
GTT	727050	16	GTC	404413	50
TAT	727013	17	CCA	355970	51
AAC	714837	18	CCT	351521	52

Symbol	Count	Identifier	Symbol	Count	Identifier
GCC	707707	19	CTC	312847	53
GCG	697732	20	TCC	288798	54
GCT	682805	21	AGA	276580	55
GGC	680436	22	CAC	261573	56
ATC	675422	23	TCG	254911	57
GAC	639516	24	CGG	223867	58
GTG	604099	25	CTA	220018	59
TTA	596272	26	TGT	210306	60
AAG	595404	27	TGC	201585	61
ACC	577696	28	CCC	193690	62
CTT	545193	29	CGA	183299	63
ACA	515145	30	AGG	157100	64
CAA	514764	31	TAA	72219	65
TTC	510621	32	TGA	56299	66
TGG	487768	33	TAG	17145	67

<unk> represents an unknown character, <pad> represents a padding character, <bos> represents a begin character, and <eos> represents an end character.

Table S3 *E. coli* codon usage frequency table

Codon	Amino	Freq	Relative Freq	Codon	Amino	Freq	Relative Freq
TTT	F	0.58	1.00	TCT	S	0.17	0.68
TTC	F	0.42	0.72	TCC	S	0.15	0.60
TTA	L	0.14	0.30	TCA	S	0.14	0.56
TTG	L	0.13	0.28	TCG	S	0.14	0.56
TAT	Y	0.59	1.00	TGT	C	0.46	0.85
TAC	Y	0.41	0.69	TGC	C	0.54	1.00
TAA	*	0.61	1.00	TGA	*	0.30	0.49
TAG	*	0.09	0.15	TGG	W	1.00	1.00
CTT	L	0.12	0.26	CCT	P	0.18	0.37
CTC	L	0.10	0.21	CCC	P	0.13	0.27
CTA	L	0.04	0.09	CCA	P	0.20	0.41
CTG	L	0.47	1.00	CCG	P	0.49	1.00
CAT	H	0.57	1.00	CGT	R	0.36	1.00
CAC	H	0.43	0.75	CGC	R	0.36	1.00
CAA	Q	0.34	0.52	CGA	R	0.07	0.19
CAG	Q	0.66	1.00	CGG	R	0.11	0.31
ATT	I	0.49	1.00	ACT	T	0.19	0.47

**Codon**	**Amino**	**Freq**	**Relative Freq**	**Codon**	**Amino**	**Freq**	**Relative Freq**
ATC	I	0.39	0.80	ACC	T	0.40	1.00
ATA	I	0.11	0.22	ACA	T	0.17	0.42
ATG	M	1.00	1.00	ACG	T	0.25	0.62
AAT	N	0.49	0.96	AGT	S	0.16	0.64
AAC	N	0.51	1.00	AGC	S	0.25	1.00
AAA	K	0.74	1.00	AGA	R	0.07	0.19
AAG	K	0.26	0.35	AGG	R	0.04	0.11
GTT	V	0.28	0.8	GCT	A	0.18	0.55
GTC	V	0.20	0.57	GCC	A	0.26	0.79
GTA	V	0.17	0.49	GCA	A	0.23	0.70
GTG	V	0.35	1.00	GCG	A	0.33	1.00
GAT	D	0.63	1.00	GGT	G	0.35	0.95
GAC	D	0.37	0.59	GGC	G	0.37	1.00
GAA	E	0.68	1.00	GGA	G	0.13	0.35
GAG	E	0.32	0.47	GGG	G	0.15	0.41

Table S4 Basic information on αGP and IA proteins

Protein	Alpha-glucan phosphorylase	Isoamylase
Short	αGP	IA
Organism	*Thermotoga maritima*	*Sulfolobus tokodaii*
EC Number	2.4.1.1	3.2.1.68
UniProt	O33831	Q973H3
GenBank	CAA04523.1	BAB65940.1
Mass（kDa）	96.139	83.054
Amino acids	822	716
